# Inherited human RelB deficiency impairs innate and adaptive immunity to infection

**DOI:** 10.1073/pnas.2321794121

**Published:** 2024-09-04

**Authors:** Tom Le Voyer, Majistor Raj Luxman Maglorius Renkilaraj, Kunihiko Moriya, Malena Pérez Lorenzo, Tina Nguyen, Liwei Gao, Tamar Rubin, Axel Cederholm, Masato Ogishi, Carlos A. Arango-Franco, Vivien Béziat, Romain Lévy, Mélanie Migaud, Franck Rapaport, Yuval Itan, Elissa K. Deenick, Irene Cortese, Andrea Lisco, Kaan Boztug, Laurent Abel, Stéphanie Boisson-Dupuis, Bertrand Boisson, Patrick Frosk, Cindy S. Ma, Nils Landegren, Fatih Celmeli, Jean-Laurent Casanova, Stuart G. Tangye, Anne Puel

**Affiliations:** ^a^Laboratory of Human Genetics of Infectious Diseases, Necker Branch, INSERM UMR 1163, Paris 75015, France; ^b^Imagine Institute, Paris Cité University, Paris 75015, France; ^c^Clinical Immunology Department, Assistance Publique Hôpitaux de Paris, Saint-Louis Hospital, Paris 75010, France; ^d^Garvan Institute of Medical Research, Darlinghurst, NSW 2010, Australia; ^e^School of Clinical Medicine, Faculty of Medicine and Health, University of New South Wales Sydney, Sydney, NSW 2052, Australia; ^f^Division of Pediatric Clinical Immunology and Allergy, Department of Pediatrics and Child Health, University of Manitoba, Winnipeg, MB R3A 1S1, Canada; ^g^Science for Life Laboratory, Department of Medical Biochemistry and Microbiology, Uppsala University, Uppsala SE-751 05, Sweden; ^h^St. Giles Laboratory of Human Genetics of Infectious Diseases, Rockefeller Branch, Rockefeller University, New York, NY 10065; ^i^Group of Inborn Errors of Immunity, Department of Microbiology and Parasitology, School of Medicine, University of Antioquia, Medellín 050010, Colombia; ^j^Department of Genetics and Genomic Sciences, Icahn School of Medicine at Mount Sinai, New York, NY 10029; ^k^Experimental Immunotherapeutics Unit, National Institute of Neurological Disorders and Stroke, NIH, Bethesda, MD 20892; ^l^Laboratory of Immunoregulation, National Institute of Allergy and Infectious Diseases, NIH, Bethesda, MD 20892; ^m^St. Anna Children’s Cancer Research Institute, Vienna 1090, Austria; ^n^Medical University of Vienna, Department of Pediatrics and Adolescent Medicine, Vienna 1090, Austria; ^o^CeMM Research Center for Molecular Medicine of the Austrian Academy of Sciences, Vienna 1090, Austria; ^p^Department of Biochemistry and Medical Genetics, Rady Faculty of Health Sciences, University of Manitoba, Winnipeg, MB R3E 0W2, Canada; ^q^Department of Allergy and Immunology, University of Medical Science, Antalya Education and Research Hospital, Antalya 07100, Türkiye; ^r^Pediatric Hematology-Immunology Unit, Necker Hospital for Sick Children, Paris 75015, France; ^s^HHMI, New York, NY 10065

**Keywords:** RelB deficiency, NF-κB pathway, autoantibodies, immunodeficiency, type I IFNs

## Abstract

We report two unrelated adults with homozygous (P1) or compound heterozygous (P2) private loss-of-function variants of V-Rel Reticuloendotheliosis Viral Oncogene Homolog B (*RELB).* The resulting deficiency of functional RelB impairs the induction of *NFKB2* mRNA and NF-κB2 (p100/p52) protein by lymphotoxin in the fibroblasts of the patients. These defects are rescued by transduction with wild-type *RELB* complementary DNA (cDNA). By contrast, the response of RelB-deficient fibroblasts to Tumor Necrosis Factor (TNF) or IL-1β via the canonical NF-κB pathway remains intact. P1 and P2 have low proportions of naïve CD4^+^ and CD8^+^ T cells and of memory B cells. Moreover, their naïve B cells cannot differentiate into immunoglobulin G (IgG)- or immunoglobulin A (IgA)-secreting cells in response to CD40L/IL-21, and the development of IL-17A/F-producing T cells is strongly impaired in vitro. Finally, the patients produce neutralizing autoantibodies against type I interferons (IFNs), even after hematopoietic stem cell transplantation, attesting to a persistent dysfunction of thymic epithelial cells in T cell selection and central tolerance to some autoantigens. Thus, inherited human RelB deficiency disrupts the alternative NF-κB pathway, underlying a T- and B cell immunodeficiency, which, together with neutralizing autoantibodies against type I IFNs, confers a predisposition to viral, bacterial, and fungal infections.

## Significance

We report two unrelated patients with combined T- and B-cell immunodeficiency and autoantibodies neutralizing type I interferons (IFNs) who developed recurrent viral, bacterial, and fungal diseases. The patients carried biallelic rare loss-of-function variants of V-Rel Reticuloendotheliosis Viral Oncogene Homolog B (*RELB)*. Their thymic stromal cells, T cells, and B cells were defective in vivo, ex vivo, and in vitro. Human RelB is essential for T- and B-cell immunity to various pathogens and for thymic stromal cell-driven tolerance to type I IFNs.

The human NF-κB family contains five structurally similar transcription factors: NF-κB1 (p105/p50), NF-κB2 (p100/p52), RelA (p65), RelB, and c-Rel. The canonical (or classical) NF-κB pathway is mediated by RelA or c-Rel together with NF-κB1 (through p50/RelA or p50/c-Rel dimers) (1), whereas RelB and NF-κB2 (through p52/RelB dimers) mediate the transcriptional activity of the alternative (noncanonical) NF-κB pathway (2). The stimulation of many upstream receptors induces a rapid but transient activation of the canonical NF-κB pathway, leading to the nuclear translocation of NF-κB dimers (1, 3). By contrast, the alternative NF-κB pathway operates with a slower, more sustained activation, and is triggered by a smaller number of ligands belonging to the Tumor Necrosis Factor (TNF) superfamily, including lymphotoxin (Lt), TWEAK, BAFF, CD40L, and RANKL (2). Activation of the noncanonical NF-κB pathway results in stabilization of the NF-κB-inducing kinase (NIK), which phosphorylates and activates IKK-α. IKK-α, in turn, induces the carboxyterminal phosphorylation of the precursor form of NF-κB2 (p100), its processing, and generation of the transcriptionally active p52/RelB heterodimer (4, 5). In the basal state, p100 inhibits transcription by forming a cytoplasmic complex with RelB, preventing the translocation of the p52–RelB complex to the nucleus (described as the IκBδ activity of p100) (4, 6).

Inborn errors of the alternative NF-κB pathway underlie immunodeficiencies, ranging from primary antibody deficiency (PAD) to combined immunodeficiency (CID) (6–9). They also affect the stromal compartment (medullary epithelial cells, mTECs), leading to the development of autoantibodies (auto-Abs) neutralizing type I interferons (IFNs) and causing susceptibility to viral infections (6, 10–12). Monoallelic variants of *NFKB2* are the most commonly described (>150 patients) inborn errors of the NF-κB pathway and three different forms of AD NF-κB2 disorders have been characterized. They are caused by LOF or GOF variants for the p52 (transcriptional activity) or IκBδ (inhibitory activity) functions of NF-κB2 (6). Patients heterozygous for p52^LOF^/IκBδ^LOF^ (p52/p100 haploinsufficiency) or p52^GOF^/IκBδ^LOF^ (p52-GOF) alleles display PAD with incomplete penetrance. Patients heterozygous for p52^LOF^/IκBδ^GOF^ alleles, the patients most frequently identified among those with AD NF-κB2 disorders, have PAD, viral diseases, pituitary endocrine defects, and/or ectodermal dysplasia (6, 13–17). AR complete NIK deficiency has been described in seven patients suffering from CID with recurrent viral, bacterial, and parasitic infections (8, 18, 19). Complete AR IKK-α deficiency causes the embryo-lethal cocoon syndrome (20), whereas AR partial IKK-α deficiency, described in four patients, can underlie syndromic ectodermal dysplasia with or without immunodeficiency (9, 21, 22).

Seven patients from three kindreds homozygous for private variants of V-Rel Reticuloendotheliosis Viral Oncogene Homolog B (*RELB)* (Y397* [*n* = 3], Q135dup [*n* = 1], P364L [*n* = 3]) have been reported (7, 23–25). These patients suffered from CID with viral [e.g., varicella-zoster virus (VZV), adenovirus], bacterial (recurrent lung infections), and/or fungal (e.g., *Talaromyces marneffei*) infections. Two of these patients also displayed autoimmune T cell infiltration of the lungs and liver (25), and five of the six tested developed auto-Abs neutralizing type I IFNs (6). The patients had normal numbers of blood monocytes, granulocytes, T, B, and NK lymphocytes, but low proportions of naïve CD45RA^+^ CD4^+^ T cells. Three related patients homozygous for the Y397* variant eventually underwent hematopoietic stem cell transplantation (HSCT) with good engraftment and immune reconstitution (26, 27). These patients probably suffered from AR RelB deficiency, but this was never proven biochemically. We aimed to clarify the contribution of RelB to human immunity by screening our database of >25,000 exomes from patients with severe infections, searching for rare predicted deleterious biallelic variants of *RELB*. We also aimed to characterize in detail the biochemical and immunological consequences of inherited RelB deficiency.

## Results

### Two Unrelated Patients with Private Biallelic Variants of *RELB*.

**Fig. 1.**
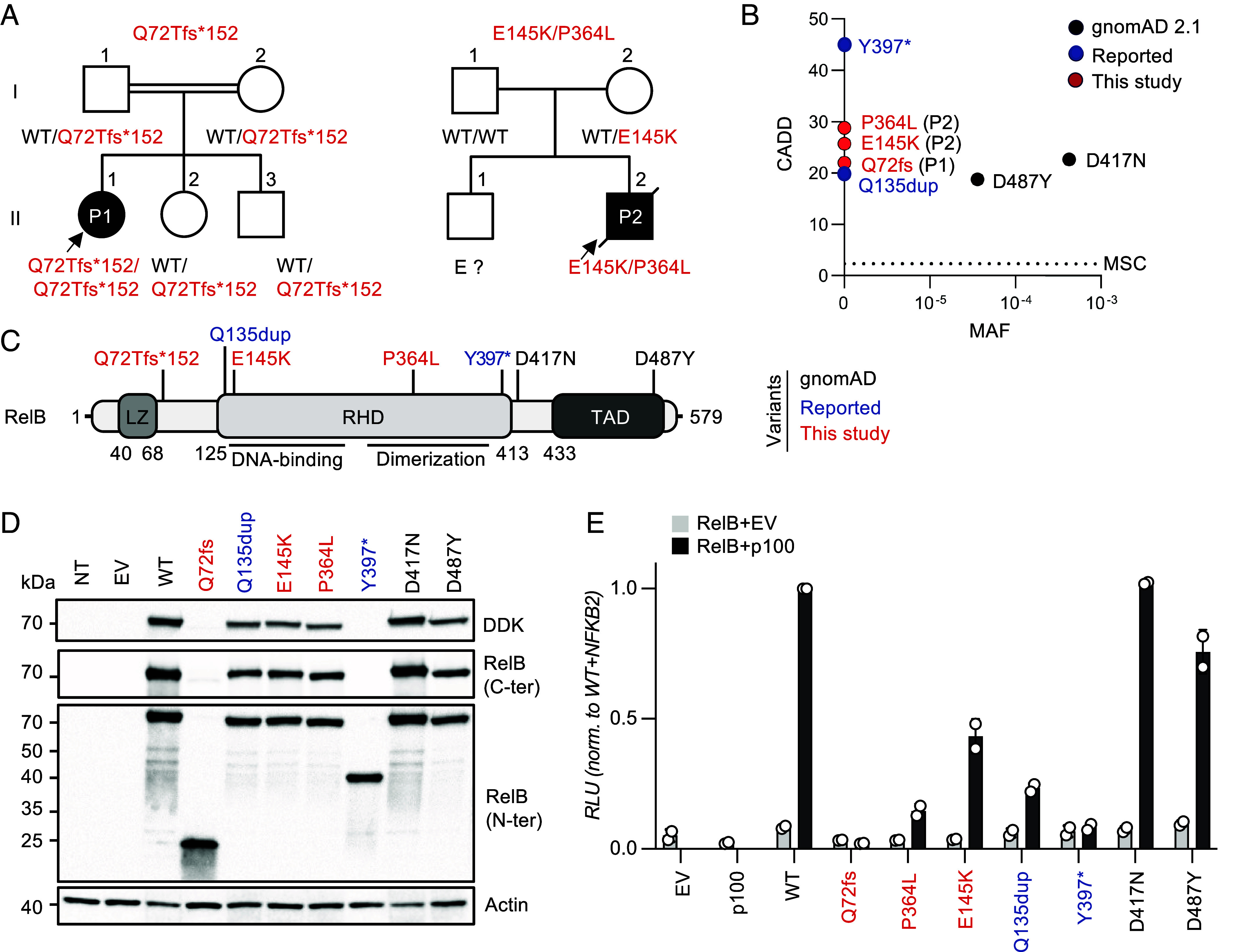
AR RelB deficiency in two unrelated patients. (*A*) Pedigrees of the two unrelated families. The familial segregation of the variants identified in P1 and P2 is depicted. The double lines connecting P1’s parents indicate consanguinity. An arrow indicates the probands. Solid symbols indicate disease status. “E?” indicates individuals of unknown genotype. (*B*) The CADD score and MAF of the variants of P1 and P2 (shown in red), the two previously reported disease-causing variants (shown in blue) (7, 23, 24), and the two homozygous missense variants found in the gnomAD 2.1 database (shown in black). All variants from P1, P2, and the four previously reported patients are private. The variants are plotted according to their CADD scores (*y*-axis) and MAF (*x*-axis). The black horizontal dotted line indicates the MSC. (*C*) Schematic diagram of the RelB protein. The regions corresponding to functionally significant domains are shown in dark gray (LZ: leucine zipper domain; RHD: Rel homology domain; TAD: transactivation domain). The variants of P1 and P2 are shown in red; the two previously reported disease-causing variants are indicated in blue, and the variants reported in the homozygous state in gnomAD appear in black. (*D*) Immunoblot analysis of RelB expression on total protein extracts from non-transfected HEK 293 T (NT) cells, or HEK 293 T cells transfected with an empty pCMV6 plasmid (EV), or pCMV6 plasmids containing the WT, P1’s variant (c.C212dup, p.Q72Tfs*152, or p.Q72fs), P2’s variant (c.433G>A/c.1091C>T, p.E145K/p.P364L), the previously reported disease-causing variants (c.1191C>A, p.Y397*, and c.400_c.401insAGC/p.Q135dup), or the two missense (c.1249G>A, p.D417N, and c.1459G>T, p.D487Y) variants of *RELB* present in the homozygous state in gnomAD. RelB was detected with a mAb specific for the C- or N-terminal region or with an antibody against the DDK tag. Actin was used as a loading control. The results shown are representative of three independent experiments. (*E*) Luciferase activity of HEK 293 T cells after 48 h of transfection with an NF-κB reporter plasmid together with an empty pCMV6 vector (EV), or a pCMV6 vector encoding wild-type (WT), P1’s variant (c.C212dup/p.Q72Tfs*152), P2’s variant (c.433G>A/p.E145K and c.1091C>T/p.P364L), the previously reported disease-causing (c.1191 C>A/p.Y397* and c.400_c.401insAGC/p.Q135dup) variants, and the two missense (c.1249 G > A/p.D417N; c.1459 G>T/p.D487Y) RelB variants present in the homozygous state in gnomAD, with or without the pCMV6*-NFKB2*-DDK plasmid encoding p100. Results were normalized against *Renilla* luciferase activity. Results were then normalized against the level of transcriptional activity for the WT p52/RelB dimer. The mean ± SD of one representative experiment (performed with a biological duplicate) is shown.

By screening our in-house database of whole-exome sequencing (WES) data for over 25,000 patients with various infectious diseases, we identified a single patient (P1) carrying a biallelic private variant of *RELB* that was predicted to be deleterious. P1 is a girl born in 2003 to consanguineous parents of Turkish origin. She had a history of recurrent oral candidiasis, facial skin lesions suspected to be of viral origin, sinusitis, multiple episodes of otitis media (leading to an episode of mastoiditis), and recurrent bacterial pneumonia. At 6 y of age, she was diagnosed with hypogammaglobulinemia, and intravenous immunoglobulin (IVIg) replacement therapy was initiated. At the age of 12 y, she was hospitalized for *Cryptococcus neoformans* meningitis (28) and CID was diagnosed. At the age of 14 y, P1 underwent successful HSCT with peripheral blood stem cells from her fully HLA-matched younger brother. She developed auto-Abs neutralizing type I IFNs 5 y after HSCT (age 19 y); no such antibodies were detected before transplantation (at 14 y of age). WES for P1 identified a homozygous single-nucleotide insertion in exon four of *RELB* (c.C212dup), introducing a frameshift, resulting in a premature stop codon: p.Q72Tfs*152 (referred to hereafter as Q72fs) (Fig. 1 *A*–*C* and *SI Appendix*, Fig. S1*A* and Table S1). Only seven other rare homozygous missense or predicted LOF variants were identified in this patient, none of which could plausibly explain her clinical phenotype (*SI Appendix*, Table S1). Sanger sequencing confirmed the homozygous nature of the frameshift variant in this patient and showed that both her parents and healthy siblings were heterozygous carriers of this variant (Fig. 1*A* and *SI Appendix*, Fig. S1*B*). Familial segregation was, therefore, consistent with AR inheritance and complete clinical penetrance (Fig. 1*A* and *SI Appendix*, Fig. S1*B*).

We recruited a second patient (P2) born to nonconsanguineous parents, of Irish, Scottish, French, and German descent, a 33-y-old man with a history of chronic mucocutaneous candidiasis (CMC), recurrent bacterial otitis, pneumonia, and peritonitis, osteomyelitis, large B cell lymphoma, chronic malabsorption syndrome with cholestatic liver disease, portal hypertension, and thyroid disease (no detectable auto-Abs against TPO or TG detected). He also suffered from severe viral infections, including varicella pneumonia requiring intensive care and mechanical ventilation at the age of 3 y, recalcitrant skin warts and epidermodysplasia verruciformis, both histologically documented. He subsequently died of JC polyomavirus-induced progressive multifocal leukoencephalitis (PML). Auto-Abs neutralizing type I IFNs were detected in serum samples collected from this patient at the ages of 32 and 34 y. P2’s parents and brother presented no unusual infectious, autoimmune, or autoinflammatory manifestations, other than chronic lymphocytic leukemia in P2’s mother. WES on P2 and his parents revealed two private heterozygous variants of *RELB*, c.433G>A and c.1091C>T, resulting in two amino acid substitutions: p.E145K and p.P364L, respectively (Fig. 1 *A*–*C* and *SI Appendix*, Tables S2 and S3). The parental linkage of P2 was confirmed by a microsatellite panel analysis. P2 harbored the maternally inherited variant c.433G>A/p.E145K and a de novo c.1091C>T/p.P364L variant.

### Private Biallelic Variants of *RELB* Segregate with Combined Immunodeficiency.

None of the three *RELB* variants identified in P1 and P2 were found in the homozygous or heterozygous state in the gnomAD 2.1 database (containing WES data from 125,748 individuals, http://gnomad.broadinstitute.org), the 1000 Genomes database (http://grch37.ensembl.org), the Single-Nucleotide Polymorphism database (dbSNP, https://www.ncbi.nlm.nih.gov/snp/), ATAVDB (http://atavdb.org), HapMap (https://www.genome.gov/10001688/international-hapmap-project), BRAVO (https://bravo.sph.umich.edu/freeze8/hg38), or in other individuals from our in-house database of 25,000 WES/WGS sequences. The combined annotation-dependent depletion (CADD) scores obtained for the *RELB* variants detected in P1 (c.C212dup, CADD = 23.8), and P2 (c.433G>A and c.1091C>T, CADD = 25.7 and 28.8, respectively) are well above the mutation significance cutoff (MSC) value of 3.313, as are the CADD scores of 45 obtained for the previously reported *RELB* variant (c.1191C>A, p.Y397*) (7, 23, 29, 30), and 18.8 obtained for the c.400_c.401insAGC/p.Q135dup *RELB* allele (24) (Fig. 1*B*). The two variants found in P2, c.433G>A (p.E145K) and c.1091C>T (p.P364L), affected two amino acids highly conserved across different species, localized in regions of the RHD involved in DNA-binding and RelB dimerization, respectively (Fig. 1*C* and *SI Appendix*, Fig. S1*C*). *RELB* has a gene damage index score of 0.59, a purifying *f* parameter of 0.32, a RVIS of −1, a LOEUF of 0.08, and a CoNeS of −1.6 (*SI Appendix*, Fig. S1 *D* and *E* and Tables S1 and S3), suggesting that this gene is under strong negative selection in human populations (31–35). Consistently, no predicted LOF (pLOF) *RELB* alleles (nonsense, frameshift, deletion/insertions, or essential splice variants) were identified in the homozygous state in gnomAD or any of the other WES/WGS databases considered. However, two individuals homozygous for rare missense *RELB* variants (c.1249 G>A, p.D417N, rs372931620, and c.1459 G>T, p.D487Y, rs144038804) were identified in gnomAD 2.1, with CADD scores of 22.7 and 18.7, respectively, and a low minor allele frequency (MAF) of 3.9 × 10^−4^ and 3.6 × 10^−5^, respectively (Fig. 1 *B* and *C*). We found no other *RELB* variant in the homozygous or compound heterozygous state in our cohort. Overall, these data strongly suggest that the patients had AR RelB deficiency and that these genotypes can account for their unusually rare and severe phenotypes.

### Mutant *RELB* Alleles Are Deleterious in an Overexpression System.

We investigated the levels of RelB protein expression from the *RELB* variant alleles identified in P1 and P2, together with the previously reported Y397* (7, 23) and Q135dup (24) *RELB* variants. We also tested the two missense *RELB* variants (D417N and D487Y) found in the homozygous state in gnomAD 2.1. Human embryonic kidney (HEK) 293T cells were transiently transfected with C-terminally DDK-tagged pCMV6 plasmids carrying the corresponding complementary DNAs (cDNAs) or an empty pCMV6 vector. All variants gave rise to amounts of mRNA similar to those for the WT *RELB* allele (*SI Appendix*, Fig. S1*F*). For the variant identified in P1 (Q72fs), and the previously reported Y397* mutant *RELB* allele (7), a monoclonal antibody (mAb) against the N-terminal region (aa ~1-100) of RelB detected a truncated protein of ~25 and ~40 kDa, respectively (Fig. 1*D*), whereas no protein was detected with an Ab against the C-terminal region of RelB, or an anti-C-terminal DDK mAb, excluding the possibility of translation reinitiation (Fig. 1*D*). Proteins of the expected molecular weight were detected for P2’s alleles (E145K and P364L), the Q135dup variant, and the two biallelic (D417N and D487Y) *RELB* variants from gnomAD (Fig. 1*D*). We then assessed these variants biochemically in an NF-κB-luciferase reporter assay in HEK 293T cells, by overexpressing the WT and mutant *RELB* cDNAs, together with a plasmid encoding the WT NF-κB2/p100 protein. The Q72fs (P1) *RELB* variant displayed no luciferase activity, like the previously reported Y397*, whereas the luciferase activity associated with the P364L and E145K alleles (the alleles present in P2) and the previously reported Q135dup *RELB* allele was impaired but not abolished (Fig. 1*E*). These data suggest that Q72fs and Y397* are amorphic, whereas Q135dup, P364L, and E145K, are strongly hypomorphic. The D417N and D487Y missense *RELB* variants, which are found in the general population, are isomorphic in this system. Collectively, these results suggest that P1 has AR complete RelB deficiency, whereas P2 has AR partial RelB deficiency. They also suggest that the patients with the previously uncharacterized Y397* variant have a complete form of AR RelB deficiency, whereas those with the previously uncharacterized Q135dup (24) and P364L variants (25) have a partial form of AR RelB deficiency.

### Abolition of RelB Expression in the Cells of P1, but Not P2.

**Fig. 2.**
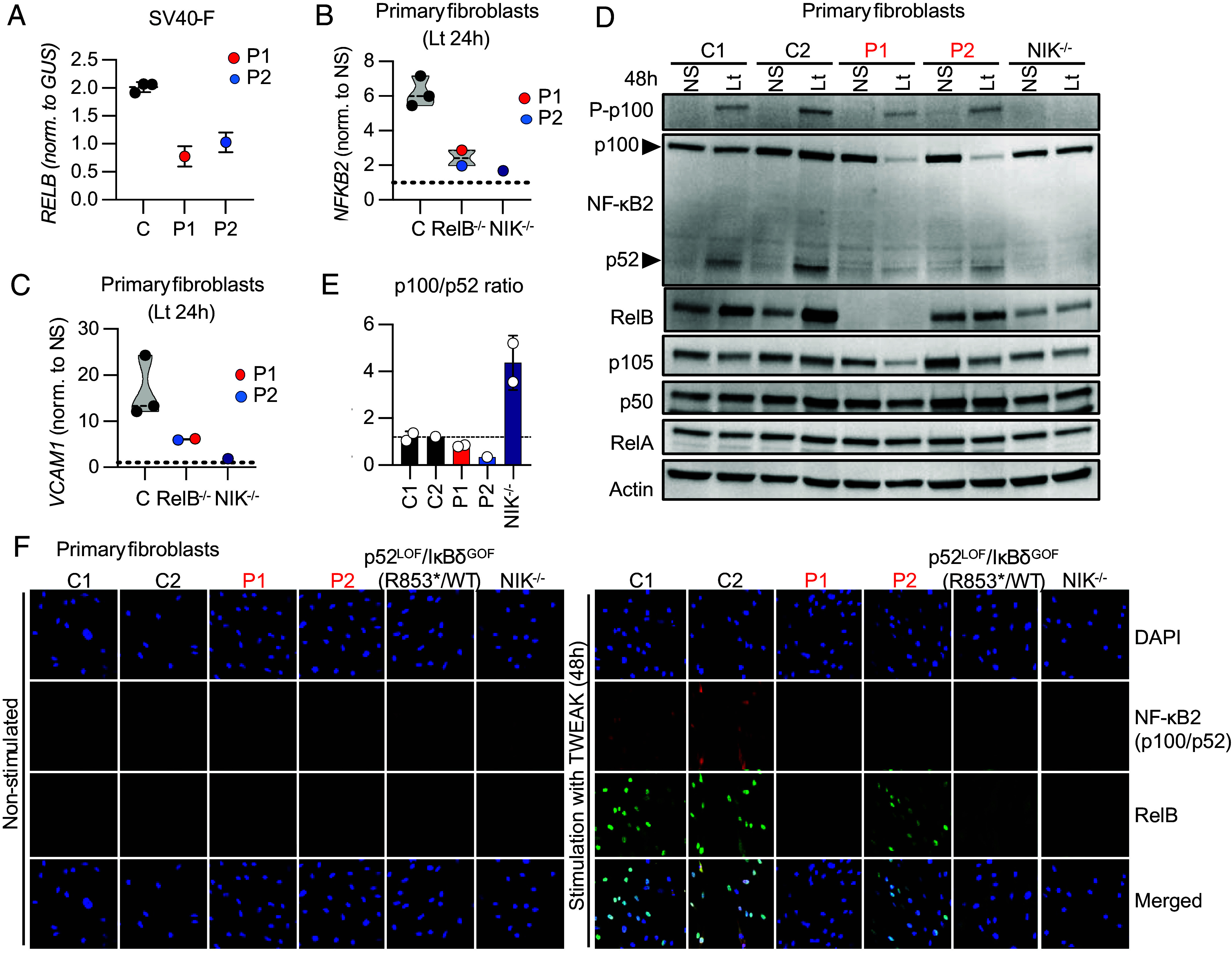
Impaired *RELB* expression and impaired activation of the alternative NF-κB pathway in the fibroblasts of P1 and P2. (*A*) RNAs extracted from SV40-immortalized fibroblasts (SV40-F) from healthy controls (C), P1, and P2 were subjected to RT-qPCR for total *RELB*. Data are expressed as 2-^Δ^*^C^*^t^ relative to the mean expression of controls, after normalization against *GUS* (endogenous control) expression (Δ*C*t). (*B* and *C*) RNA extracted from primary fibroblasts from three different healthy subjects (*C*), P1, P2, and a patient with AR complete NIK deficiency stimulated with lymphotoxin α1β2 (Lt) for 24 h and subjected to RT-qPCR for *NFKB2* (*B*) or *VCAM1* (*C*). (*D*) Immunoblot analysis of phosphorylated p100 (P-p100), NF-κB2 (p100/p52), RelB, NF-κB1 (p105/p50), and RelA levels in primary fibroblasts from healthy controls (C1 and C2), P1, P2, and a NIK-deficient patient (NIK^−/−^) (8). Actin immunoblotting was used as a loading control. The results shown are representative of two independent experiments. (*E*) Graph showing the p100/p52 ratio in primary fibroblasts from healthy controls (C1 and C2), P1, P2, and a NIK-deficient patient (NIK^−/−^), based on data from two independent experiments. (*F*) Primary fibroblasts from controls (C1 and C2), the two RelB-deficient patients (P1 and P2, in red), a patient with a p52^LOF^/IκBδ^GOF^ variant (6) and a NIK-deficient patient (NIK^−/−^) (8), were left unstimulated or were stimulated with TWEAK for 48 h and stained with a rabbit anti-C-terminal RelB or a mouse anti-N-terminal p100 mAb. Nuclei were stained with DAPI.

We evaluated *RELB* transcript levels by RT-qPCR in EBV-B cells from P1 and P1’s heterozygous mother, and in SV40-immortalized fibroblasts (SV40-F) from P1, P2, and a NIK-deficient patient (8). *RELB* transcript levels were very low in P1’s EBV-B cells, whereas the *RELB* transcript levels in the EBV-B cells from her heterozygous mother were intermediate between those of P1 and those of healthy subjects (*SI Appendix*, Fig. S2*A*). *RELB* transcript levels were also lower in SV40-F from P1 and P2 than in healthy control and NIK-deficient SV40-F (Fig. 2*A*). No full-length transcripts were detected by RT-PCR in P1’s EBV-B cells, suggesting that c.C212dup *RELB* transcripts were degraded by nonsense-mediated mRNA decay (*SI Appendix*, Fig. S2*B*). No RelB protein was detected in EBV-B cells (*SI Appendix*, Fig. S2*C*) or SV40-F (*SI Appendix*, Fig. S2*D*) from P1, on immunoblots with the anti-C-terminal RelB mAb. Cells heterozygous for the Q72fs *RELB* variant contained less than half the amount of RelB detected in cells from healthy subjects (*SI Appendix*, Fig. S2 *C* and *D*). Similarly, NIK-deficient SV40-F had lower levels of RelB than control cells (*SI Appendix*, Fig. S2*D*). By contrast, RelB levels in primary fibroblasts from P2 were similar to those in primary fibroblasts from healthy donors (Fig. 2*D*). Overall, these results suggest that the c.C212dup *RELB* variant abolishes RelB expression in both leukocytic and nonleukocytic cells of the patient. By contrast, the E145K and P364L variants identified in P2 encode a mutant RelB protein produced at levels similar to those of the WT protein in healthy donors.

### Impaired Activation of the Alternative NF-κB Pathway in *RELB* Mutant Fibroblasts.

We evaluated the activation of the alternative NF-κB pathway in primary and SV40 fibroblasts from P1 and P2. We assessed *NFKB2* mRNA induction, and the production, phosphorylation, and processing of p100 into p52 upon stimulation with lymphotoxin (Lt) or TWEAK (6). *NFKB2* induction by Lt or TWEAK was strongly impaired, as shown by RT-qPCR, in cells from P1 and P2 relative to fibroblasts from healthy subjects, whereas this induction was abolished in NIK-deficient fibroblasts, due to the lack of p52/RelB heterodimer formation (8) (Fig. 2*B* and *SI Appendix*, Fig. S2*E*). After 48 h of Lt stimulation, the phosphorylation of p100 in primary fibroblasts from P1 and P2 was similar to that in control fibroblasts, whereas it was abolished in NIK-deficient fibroblasts (Fig. 2*D*). However, the amount of unprocessed p100 following Lt stimulation was much smaller in the primary fibroblasts of P1 and P2 than in control or NIK-deficient primary fibroblasts. Furthermore, p52 levels following Lt stimulation were also lower in the cells of P1 and P2 than in healthy donor cells, whereas p52 was undetectable in NIK-deficient cells. However, no defect in the processing of p100 to generate p52 was detected in the cells of P1 and P2, as shown by the similar p100/p52 ratios of patient and control cells (Fig. 2*E*). Confocal microcopy on primary or SV40 fibroblasts showed that RelB was normally translocated to the nucleus following the stimulation of cells from P2 with TWEAK (Fig. 2*F* and *SI Appendix*, Fig. S2*F*). Consistent with an impairment of RelB-dependent *NFKB2* induction after activation of the alternative NF-κB pathway, primary and SV40 fibroblasts from P1 and P2, a patient heterozygous for a p52^LOF^/IκBδ^GOF^ *NFKB2* variant (6), and an NIK-deficient patient contained less nuclear p52 than control fibroblasts after TWEAK stimulation (Fig. 2*F* and *SI Appendix*, Fig. S2*F*). An impairment of alternative NF-κB pathway activation was confirmed in primary fibroblasts from both P1 and P2, with impaired upregulation of *VCAM1*, a known target of the p52/RelB heterodimer (36), after Lt stimulation (Fig. 2*C*). Together, these results suggest that RelB plays a nonredundant role in activating the alternative NF-κB pathway after Lt and TWEAK stimulation, impairing RelB/p52 dimer formation, without affecting the processing of p100 to generate p52.

### Rescue of Defective Alternative NF-κB Signaling in P1 and P2 Fibroblasts.

**Fig. 3.**
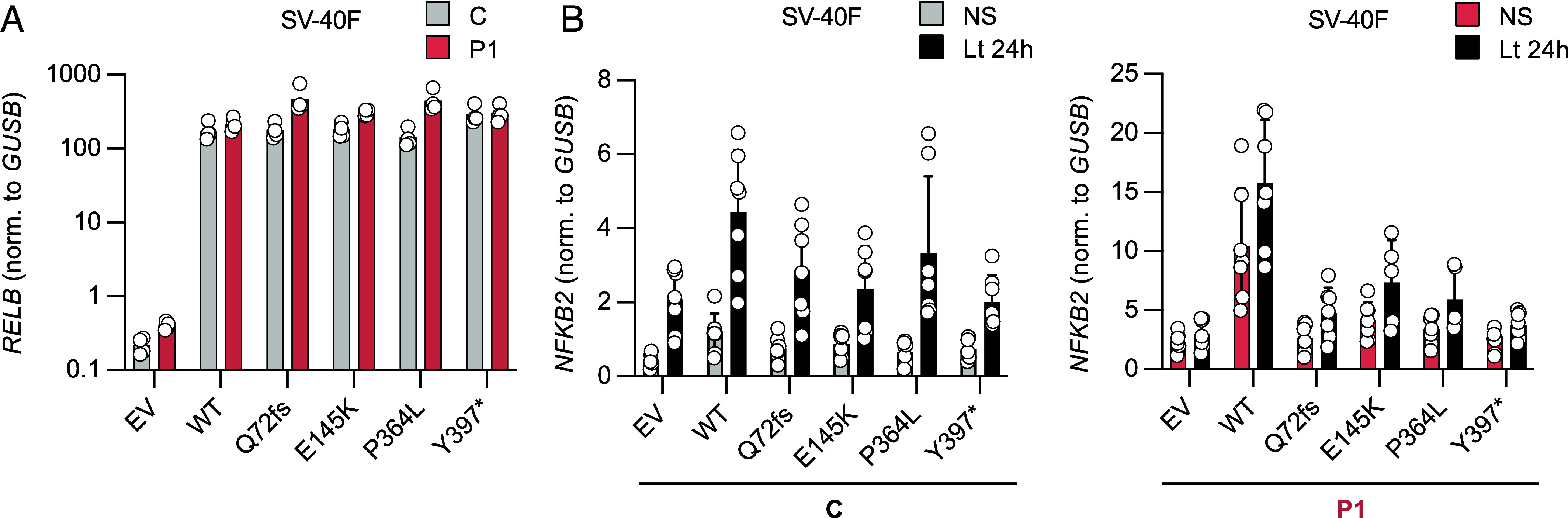
Rescue of defective non-canonical NF-κB signaling in P1 fibroblasts. (*A*) SV40-F from a healthy control (C) and P1 were stably transduced with an empty vector (EV) or the WT *RELB* cDNA. SV40-F from the healthy control and P1 were also transduced with the Q72Tfs, E145K, P364L, or Y397* mutant *RELB* cDNA. The RNA extracted from these cells was subjected to RT-qPCR for total *RELB*. Data are displayed as 2^−ΔCt^ relative to EV-transduced cells, after normalization against *GUSB* (endogenous control) expression (ΔCt). The result of one representative experiment is shown. (*B*) SV40-F from a healthy control (*Left*) or from P1 (*Right*) were stably transduced with an EV, the WT or a mutant (Q72fs, E145K, P364L, or Y397*) *RELB* cDNA. The cells were either left unstimulated (NS, gray bars for the control or red bars for P1) or were stimulated for 24 h with Lt (black bars). The RNA extracted from these cells was subjected to RT-qPCR for total *NFKB2*. Data are displayed as 2^−ΔCt^ values relative to unstimulated EV-transduced cells, after normalization against *GUSB* (endogenous control) expression (ΔCt). The result of one independent experiment is shown.

We attempted to rescue the patients’ cellular phenotype by reintroducing the WT *RELB* cDNA by stable lentiviral transduction into SV40-F from P1 and P2. We used an empty pTRIP-SFFV-ΔNGFR-2A lentivirus (EV), or a pTRIP-SFFV-ΔNGFR-2A lentivirus encoding the WT, P1 (Q72fs), P2 (E145K or P364L), the previously reported (Y397*) *RELB* variants, or a WT *MAP3K14* cDNA encoding NIK. Healthy donor (C) and P1 SV40-F transduced with any of the five *RELB* cDNAs, and P2 SV40-F transduced with WT *RELB* cDNA produced similar amounts of *RELB* mRNA (Fig. 3*A* and *SI Appendix*, Fig. S2*G*). By contrast, the WT *RELB* cDNA, unlike the mutants tested, fully restored *NFKB2* mRNA induction after 24 h of stimulation with Lt in P1’s SV40-F, whereas the transduction of control SV40-F with any *RELB* allele or EV resulted in similar levels of *NFKB2* induction (Fig. 3*B* and *SI Appendix*, Fig. S2*H*). WT *RELB* restored *NFKB2* induction in transduced P2 SV40-F, whereas transduction with the EV did not. Similar results were obtained for NIK-deficient fibroblasts transduced with a plasmid encoding WT NIK (*SI Appendix*, Fig. S2*I*). Overall, these data confirm that inherited RelB deficiency impairs activation of the alternative NF-κB pathway.

### Normal Activation of the Canonical NF-κB Pathway in *RELB*-Deficient Fibroblasts.

**Fig. 4.**
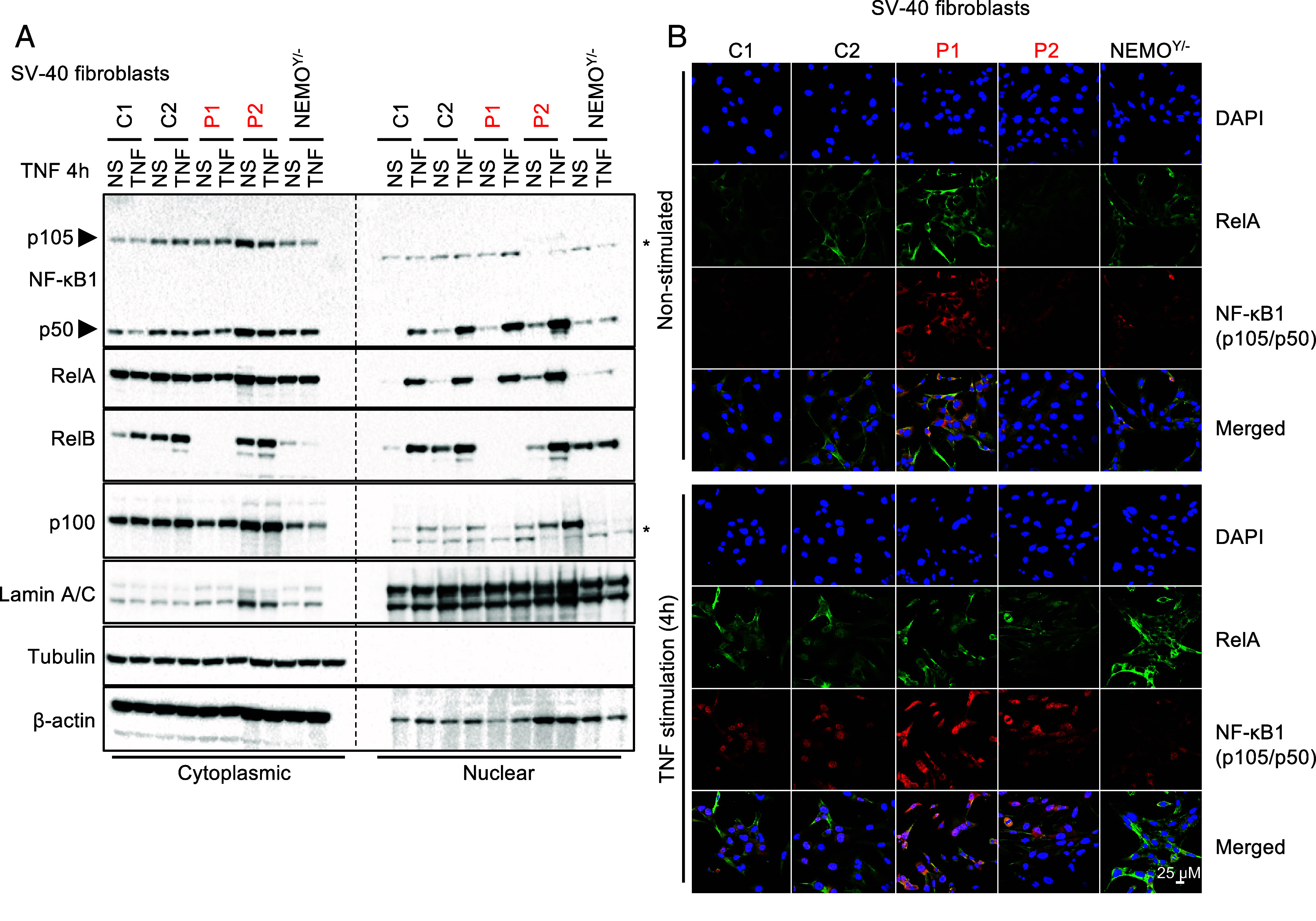
Normal activation of the canonical NF-κB pathway in fibroblasts from P1 and P2. (*A*) Immunoblot analysis of the cytoplasmic and nuclear fractions of SV40-F from healthy controls (C1 and C2), P1, P2, or SV40-F deficient for NEMO (NEMO^Y/−^) left unstimulated or stimulated with TNF for 4 h, and assessed for the levels of NF-κB1 (p105/p50), RelA (p65), RelB, and NF-κB2 (p100). The (*) symbol indicates non-specific bands. Tubulin and β-actin were used as loading controls. (*B*) SV40-F from controls (C1 and C2), the two RelB-deficient patients (P1 and P2, in red), and NEMO-deficient SV40-F (NEMO^Y/−^) were stimulated with TNF for 4 h, and stained with a mouse anti-C-terminal RelA or a mouse anti-N-terminal p105 antibody. Nuclei were labeled with DAPI.

We also assessed the activation and functionality of the canonical NF-κB pathway in primary and SV40-transformed fibroblasts from P1 and P2. P1’s SV40-fibroblasts had normal levels of NF-κB1 (p105/p50) and RelA and, unlike NEMO-deficient SV40 fibroblasts, displayed normal IκB-α degradation in response to TNF or IL-1β (*SI Appendix*, Fig. S3*A*). Upon TNF stimulation, p50 and RelA were translocated to the nucleus to similar levels in SV40-F from both patients and healthy donors (Fig. 4 *A* and *B*). In addition, SV40-F from P2 displayed normal RelB nuclear translocation in response to TNF stimulation, whereas no RelB was detected in the cells of P1. Normal levels of unprocessed p100 were observed following TNF stimulation in SV-40 fibroblasts from P1 and P2, but not in NEMO-deficient fibroblasts, suggesting that IκBδ activity was normal in the patients (Fig. 4*A*). Finally, we assessed IL-6 production after stimulation with TNF, IL-1β, and various TLR agonists. P1’s SV40-F responded similarly to control cells, whereas IRAK4-deficient fibroblasts (37) were unresponsive to TLR/IL-1R (TIR) agonists (except poly:IC) (*SI Appendix*, Fig. S3*B*). Collectively, these results indicate that the deleterious *RELB* variants identified in P1 and P2 impair the induction of target genes controlled by p52/RelB heterodimer activation via the alternative NF-κB signaling pathway, without affecting the p50- and RelA-dependent dimers involved in canonical NF-κB signaling pathway.

### Neutralizing Auto-Abs against Type I IFNs in Patients with AR RelB Deficiency.

**Fig. 5.**
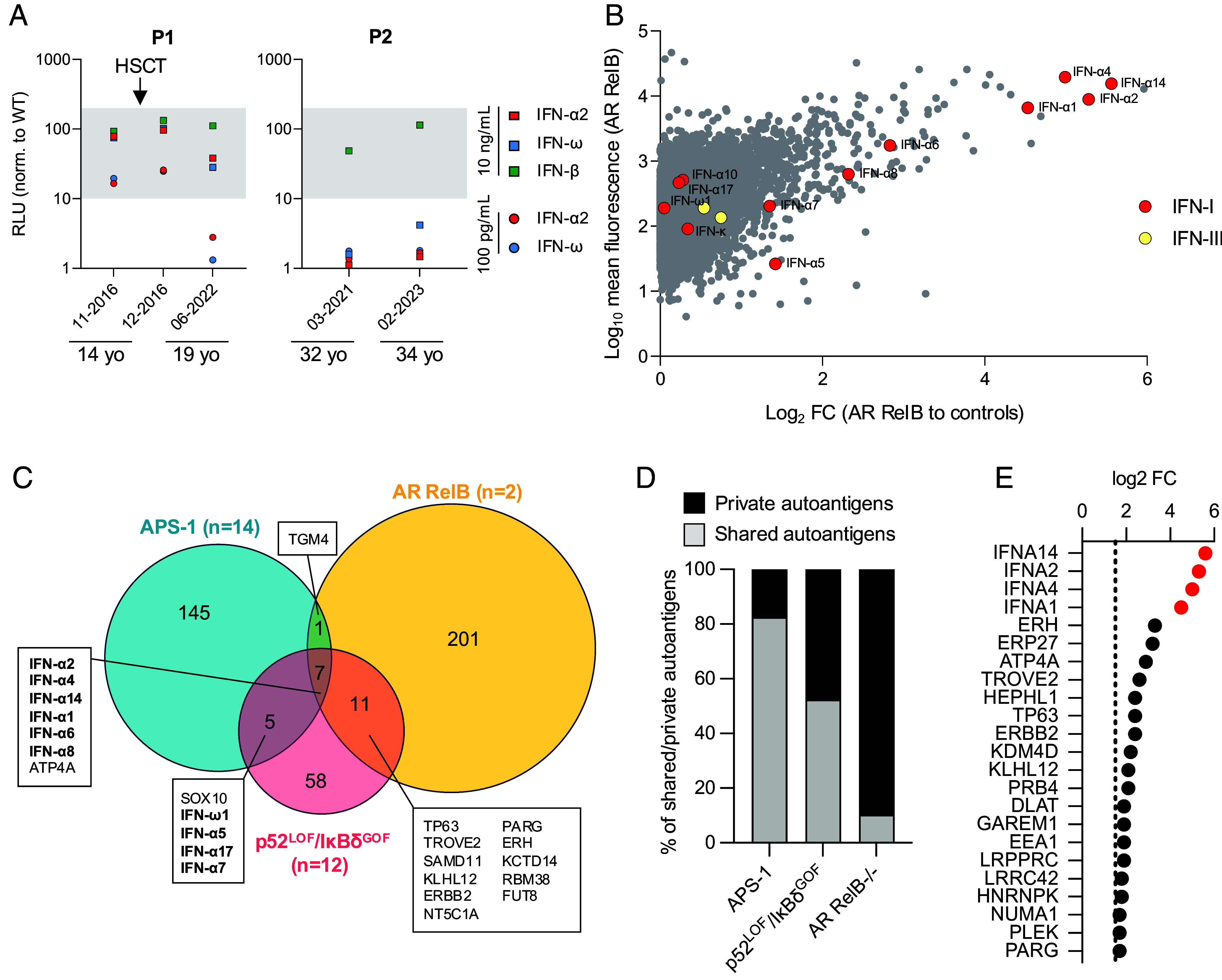
Auto-Abs against type I IFNs in P1 and P2. (*A*) Auto-Abs neutralizing IFN-α2, IFN-ω, or IFN-β at concentrations of 10 ng/mL or 100 pg/mL were assessed in an ISRE luciferase assay. The gray area corresponds to the normal ISRE activity obtained with non-neutralizing plasma from healthy controls. (*B*) Protein microarray showing the distribution of auto-Ab reactivity against 20,000 human proteins in plasma samples from patients with AR RelB deficiency (*n* = 2, P1 and P2, aged 19 and 32 y, respectively). Data are expressed as the fold-change relative to 22 plasma samples from healthy donors. Red dots represent type I IFNs and yellow dots represent type III IFNs. Data for HuProt and neutralization assay experiments are presented as the mean for at least two technical replicates. (*C*) Venn diagram showing the enriched autoantigen profile (log_2_-fold change relative to healthy controls >1.5) of patients with APS-1 (*n* = 14), p52^LOF^/IκBδ^GOF^ variants (*n* = 12), or AR RelB deficiency (*n* = 2). Type I IFNs are indicated in bold. (*D*) Proportion of shared (by ≥2 patients) and private reactive autoantigens in the group of patients with APS-1, a p52^LOF^/IκBδ^GOF^ variant, or AR RelB deficiency. (*E*) Reactive autoantigens common to the two patients with AR RelB deficiency included in the analysis. Red dots indicate type I IFNs.

The human alternative NF-κB pathway is essential for thymic medullary organization, mTEC development, and AIRE expression (6). At the age of 14 y (before transplantation) the thymus from P1 was strongly hypoplastic, consistent with findings for patients heterozygous for p52^LOF^/IκBδ^GOF^ *NFKB2* variants and previously reported patients with AR partial or complete RelB deficiency (24). Most patients with inborn errors of the alternative NF-κB pathway have auto-Abs neutralizing type I IFNs conferring a predisposition to severe viral diseases (6, 38). We recently reported the presence of auto-Abs neutralizing type I IFNs in the plasma of P1 and P2 (6). Consistent with the essential role of p52/RelB heterodimers in nonhematopoietic mTEC development, these neutralizing auto-Abs appeared 5 y after HSCT in P1 and were not detectable before HSCT (Fig. 5*A*). We searched for auto-Abs against other proteins in plasma samples from P1 and P2, using a panel of about 20,000 full-length human proteins (the Human Proteome Array, HuProt). According to HuProt, type I IFNs were the autoreactive antigen displaying the highest level of enrichment in patients with AR RelB deficiency relative to healthy controls (Fig. 5*B* and *SI Appendix*, Fig. S4). We then compared the overlap of autoreactive antigen enrichment (log_2_-fold change relative to healthy controls >1.5 in each group) between patients with AR RelB (*n* = 2), autoimmune polyglandular syndrome type 1 (APS-1, *n* = 14) and p52^LOF^/IκBδ^GOF^ variants (*n* = 12), as in a previous study (6). RelB-deficient patients had larger numbers of enriched autoreactive antigens (*n* = 220) than APS-1 patients (*n* = 158) or patients heterozygous for p52^LOF^/IκBδ^GOF^ variants (*n* = 82) (Fig. 5*C*). This apparently broader autoreactive profile may be explained by the smaller number of RelB-deficient patients included, as most of the reactive antigens were private to the two patients analyzed (Fig. 5*D*). Six of the seven autoreactive antigens displaying enrichment in all three groups of patients were type I IFNs (Fig. 5*E*). Very few other APS-1 autoantigens were common to the lists of autoantigens for p52^LOF^/IκBδ^GOF^ patients (SOX10), or RelB-deficient patients (TGM4). In addition, only 11 autoreactive antigens were common to patients with p52^LOF^/IκBδ^GOF^ and patients with RelB variants (Fig. 5*C*). Type I IFNs were the most highly enriched autoreactive antigens common to these two inborn errors of the alternative NF-κB pathway (33%, *n* = 6/18 of the shared autoantigens). Overall, these data suggest that RelB is essential for thymic T cell tolerance to type I IFNs.

### RelB Deficiency Compromises T Cell Development and Maturation.

**Fig. 6.**
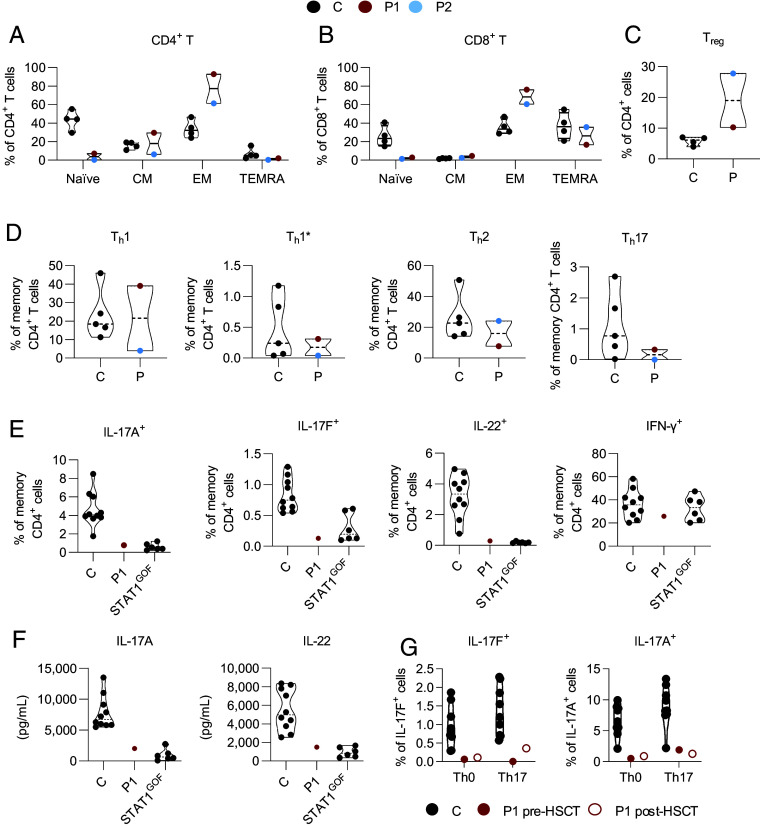
RelB deficiency compromises T cell development and functions. Immunophenotyping of PBMCs from 4-13 healthy controls (C, black dots), P1 (red dots), and P2 (blue dots). (*A*–*C*) Frequency of naïve (CD45RA^+^CCR7^+^), central memory (CM, CD45RA^−^CCR7^+^), effector memory (EM, CD45RA^+/−^CCR7^−^), and terminally differentiated effector memory (TEMRA, CDR45RA^−^CCR7^−^) cells among the CD4^+^ (*A*) and CD8^+^ (*B*) T cells of healthy subjects (*C*, *n* = 4), P1 before HSCT, and P2. (*C*) Frequency of T_reg_ (CD3^+^CD4^+^CD25^hi^FoxP3^+^) cells in the CD4^+^ T cell compartment in controls (*n* = 4), P1 before HSCT, and P2. (*D*) Frequencies of T_h_ subsets among memory CD4^+^ T cells from controls (*n* = 5), P1 before HSCT, and P2. Subsets were defined as follows: T_h_1 (CXCR5^−^CXCR3^+^CCR4^−^CCR6^−^), T_h_1* (CXCR5^−^CXCR3^+^CCR4^−^CCR6^+^), T_h_2 (CXCR5^−^CXCR3^−^CCR4^+^CCR6^−^), and T_h_17 (CXCR5^−^CXCR3^−^CCR4^+^CCR6^+^). Dotted horizontal lines represent the median value in each distribution. (*E*) Percentages of cells expressing IL-17A, IL-17F, IL-22, and IFN-γ ex vivo, as determined by flow cytometry, among memory CD4^+^ T cells from PBMCs activated by incubation with PMA and ionomycin for 8 h. (*F*) IL-17A and IL-22 production assessed by specific ELISA on whole-blood supernatants stimulated by incubation for 24 h with PMA and ionomycin. The two experiments were conducted in parallel for healthy subjects (*n* = 10), the RelB-deficient patient (P1), and patients with heterozygous gain-of-function *STAT1* mutations (*n* = 6). (*G*) Cytokine production, measured by ELISA, for IL-17A and IL-17F after 4 d of culture in T_h_0 or T_h_17 conditions, for cells from nine different healthy donors, and from P1 before (pre-) and after (post-) HSCT.

Polymorphonuclear (neutrophil and basophil) leukocyte and monocyte counts were within the normal ranges for P1 and P2 (*SI Appendix*, Tables S4 and S5). We used flow cytometry to evaluate various leukocyte subsets in PBMCs from P1 and P2. The proportions of cDC1 and mDC were lower than those in healthy donors; cDC2 proportions were normal, and pDCs were slightly more abundant in both patients (*SI Appendix*, Fig. S5*A*). The frequencies of total innate lymphoid cells (ILC), ILC progenitors (ILCP), and ILC2 were within the ranges found in healthy subjects (*SI Appendix*, Fig. S5*A*). NK cell counts and proportions were within the normal range in both patients, with proportions of CD56^bright^ similar to (P2) or higher than (P1) those in healthy donors (*SI Appendix*, Fig. S5*A* and Tables S4 and S5). For other innate-like lymphocyte subsets, we found that both patients had proportions of iNKT at the lower end of the range for healthy donors, lower proportions of MAIT cells, and normal proportions of γδ T cells (*SI Appendix*, Fig. S5*A*). Counts of CD3^+^, CD4^+^, and CD8^+^ T cells were normal in P1 (assessed at the age of 12 y), but markedly low in P2 (assessed at the age of 19 y), except for CD8^+^ T cells (308, 168, and 132 cells/mm^3^, respectively) (*SI Appendix*, Tables S4 and S5). P1 had low T cell receptor excision circle (TREC) levels (*SI Appendix*, Table S4; not assessed in P2). P1 and P2 had low proportions of recent thymic emigrant cells (*SI Appendix*, Tables S4 and S5), low frequencies of naïve CD4^+^ and CD8^+^ T cells, and high frequencies of effector memory CD4^+^ and CD8^+^ T cells (*SI Appendix*, Tables S4 and S5; Fig. 6 *A* and *B*). The proportions of central memory CD4^+^ and CD8^+^ T cells in both patients remained within the range for healthy donors (Fig. 6 *A* and *B* and *SI Appendix*, Tables S4 and S5). Furthermore, a study of P2’s TCR Vβ repertoire showed a greater representation of TCR Vβ21.3, with a concomitant decrease in the other TCR Vβ families, suggesting a peripheral expansion of specific T cell clones. Ex vivo analysis of T cell subsets revealed high proportions (P1 and P2), but low numbers (P2) of T_reg_ and cT_fh_ (Fig. 6*C* and *SI Appendix*, Fig. S5 *B* and *C* and Tables S4 and S5), normal proportions of T_h_1, T_h_1*, and T_h_2, and slightly lower proportions of T_h_17 (Fig. 6*D*) cells. Overall, these results suggest that partial or complete forms of AR RelB deficiency caused inadequate thymopoiesis, defects of T lymphocyte development, maturation, and differentiation.

### RelB Deficiency Compromises T Cell Functions.

Both RelB-deficient patients experienced infections related to a T cell defect: CMC (P1 and P2), *C. neoformans* meningitis (P1), epidermodysplasia verruciformis and recalcitrant skin warts (P2), and JC virus-induced PML (P2). We therefore analyzed the impact of RelB deficiency on T cell function. T lymphocyte proliferation was compromised in P2, reaching levels 9 to 40% of that in healthy donors in response to mitogens (pokeweed mitogen [PWM], phytohemagglutinin [PHA], concavalin A [ConA]), and antigens (*Candida*, VZV) (*SI Appendix*, Table S6). Under T_h_0 or T_h_1 polarizing conditions, P1’s naïve CD4^+^ T cells displayed an intact upregulation of the expression of activation markers (CXCR5, CD40L, PD-1, CD25, CD69, ICOS) (*SI Appendix*, Fig. S6). We then assessed cytokine production by CD4^+^ T cells in response to PMA/ionomycin stimulation. Like patients with STAT1 GOF (39, 40), P1 had low proportions of IL-17A-, IL-17F-, and IL-22- producing, but not of IFN-γ-producing, memory CD4^+^ T cells ex vivo (Fig. 6*E*). Similarly, P1 and STAT1 GOF whole-blood cells secreted less IL-17A and IL-22 than cells from healthy subjects following PMA/ionomycin stimulation (Fig. 6*F*). We then assessed P1’s sorted memory CD4^+^ T cells under T_h_0, T_h_1, and T_h_17 polarizing conditions, before and after HSCT. Before HSCT, the proportion of IL-2-expressing memory CD4^+^ T cells was normal under T_h_0 conditions, but IL-2 production was impaired in P1 (*SI Appendix*, Fig. S7 *A* and *B*). The proportions of TNF- and IFN-γ-producing memory CD4^+^ T cells were unaffected and high, respectively (*SI Appendix*, Fig. S7*A*), consistent with the detection of a higher proportion of T_h_1-type memory cells by flow cytometry in P1 (Fig. 6*D*). However, these cells secreted smaller amounts of these cytokines than memory CD4^+^ T cells from healthy donors (*SI Appendix*, Fig. S7*B*). In addition, before HSCT, the proportions of IL-4-, IL-9-, IL-10-, IL-21-, IL-17A-, IL-17F-, and IL-22-expressing cells among memory CD4^+^ T cells were low (Fig. 6*G* and *SI Appendix*, Fig. S7*A*). Similarly, only very small amounts of IL-4, IL-5, IL-9, IL-10, IL-13, IL-17A, IL-17F, and IL-22 were secreted by memory CD4^+^ T cells (*SI Appendix*, Fig. S7*B*). Six months after HSCT, most of these defects in cytokine production by memory CD4^+^ T cells observed ex vivo had been corrected, with the exception of IL-17A and IL-17F production, which remained persistently low (Fig. 6*G*). A reconstitution of the naïve T cell population is generally observed 9 mo after HSCT, but may take about 24 mo for memory T cells (41, 42). Thus, despite the detection of only small proportions of IL-17A/F-producing cells 6 mo after HSCT, P1 remained free from CMC and other fungal diseases 3 y after HSCT (*Materials and Methods*). These results suggest that the RelB deficiency compromised the differentiation of naïve CD4^+^ T cells into effector cytokine-producing memory CD4^+^ T cells, consistent with the cytokine production defect documented ex vivo in memory CD4^+^ T cells from P1. They also suggest that the observed T_h_17 defect (in terms of cell proportions and cytokine production) probably accounted for CMC in both RelB-deficient patients.

### RelB Deficiency Impairs Peripheral B Cell Development and Function.

**Fig. 7.**
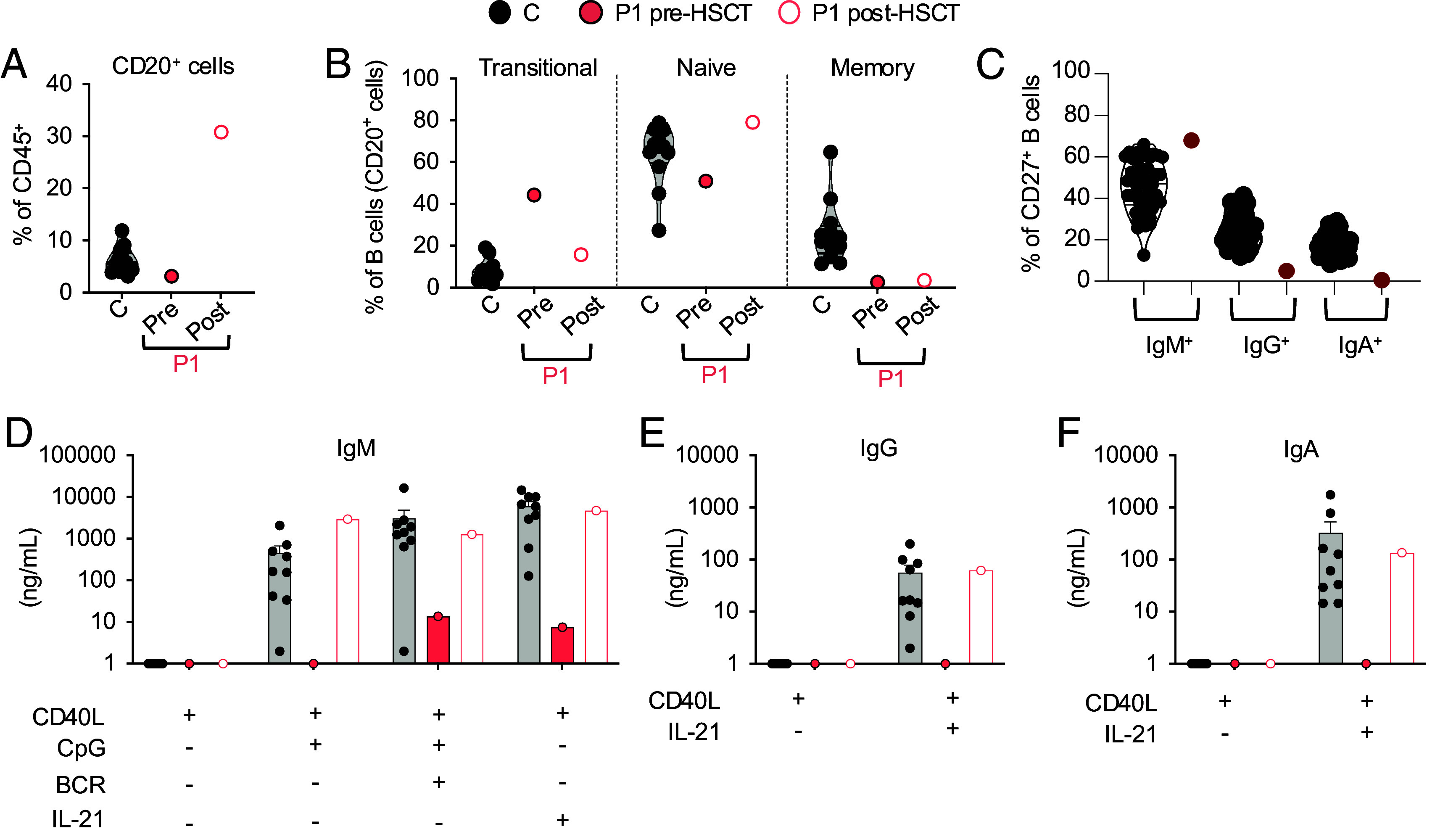
RelB deficiency impairs peripheral B cell development and function in P1. (*A* and *B*) Immunophenotyping of PBMCs from healthy donors (*C*, *n* = 10, filled black circles), and P1 before (pre, filled red circles) and after (post, red circles) HSCT. Subsets were defined by labeling with mAbs against CD20, CD10, CD27, IgG, IgA, and IgM. The proportions of B (CD20^+^) cells within the lymphocyte gate (*A*), and of transitional (IgM^+^IgD^+/−^CD10^+^CD27^−^), naïve (IgM^+^IgD^+^CD27^−^), and memory (CD27^+^) cells within the B cell compartment (*B*) are shown. (*C*) Proportions of IgM^+^, IgG^+^, and IgA^+^ cells within the memory B cell population, as determined by flow cytometry. (*D*–*F*). Naïve B cells from six healthy subjects, and P1 before (pre) and after (post) HSCT were sorted and incubated with CD40L alone or together with CpG, BCR ligand, or IL-21 for 7 d. The secretion of IgM (*D*), IgG (*E*), and IgA (*F*) into the supernatant was assessed by Ig heavy chain–specific ELISA. The limit of detection corresponds to 1.

Both patients suffered from recurrent respiratory tract infections, otitis media (complicated by an episode of mastoiditis in P1), and repeated bacterial pneumonia, suggestive of a severe B cell defect. Given the high levels of RelB expression across all B cell subsets, we investigated the impact of RelB deficiency on B cell development and function. Total B lymphocyte counts and proportions were normal in P1 at the age of 12 y (pre-HSCT) (*SI Appendix*, Table S4 and Fig. 7*A*), but were low (56/mm^3^, normal range: 79 to 574/mm^3^) in P2 at the age of 19 y (pre-rituximab treatment) (*SI Appendix*, Table S5). However, P1 had a higher proportion of transitional B cells, normal proportions of naive B cells, and a lower proportion of memory B cells than healthy donors (Fig. 7*B*). Total B cell proportions increased after HSCT, to the high levels observed in the peripheral blood of neonates, but memory B cell levels remained at the lower end of the range for healthy donors 6 mo post-HSCT (Fig. 7 *A* and *B* and *SI Appendix*, Table S4). This finding is consistent with the kinetics of memory B cell reconstitution, a process occurring over a period of 12 to 24 mo after HSCT (43, 44). In healthy donors, the memory B cell pool contains 15 to 40% IgG^+^ cells, and 10 to 25% IgA^+^ cells, with about 50% of cells remaining unswitched and continuing to express IgM (Fig. 7*C*) (45). By contrast, <5% of the residual memory B cells detected in P1 before HCST had undergone class switching in vivo to express immunoglobulin G (IgG) or immunoglobulin A (IgA). Indeed, >70% remained IgM^+^ (Fig. 7*C*). We were unable to evaluate antigen-specific antibody (Ab) responses before IVIg replacement in P1, but P2 had inadequate specific Ab responses following vaccination against tetanus, diphtheria, and measles/mumps/rubella (MMR), and VZV, despite having had chicken pox and shingles due to VZV infection (*SI Appendix*, Table S5 and *Materials and Methods*). In vitro, the proliferation of naïve B cells isolated from P1 before HSCT in response to CD40L, alone or in the presence of CpG, anti-IgM antibody, or BAFF, was similar to that of healthy donor naïve B cells, as demonstrated by CFSE dilution (*SI Appendix*, Fig. S8*A*). However, proliferation rates decreased slightly in response to stimulation with CD40+IL-21 (*SI Appendix*, Fig. S8*A*). Like proliferation, the upregulation of HLA-DR, CD80, CD86, CD95, and IL-21R on naïve RelB-deficient B cells activated in vitro was globally intact (*SI Appendix*, Fig. S8*B*). In stark contrast, the induction of IgM secretion by P1’s naïve B cells collected pre-HSCT and stimulated with CD40L in combination with CpG, CpG plus BCR agonist, or IL-21 was barely detectable, contrasting with results obtained for naïve B cells from healthy subjects (Fig. 7*D*). Furthermore, RelB-deficient naïve B cells were unable to undergo class switching to secrete IgG or IgA in vitro in response to CD40L+IL-21 (Fig. 7 *E* and *F*), consistent with the low levels of serum IgG and IgA in P1 before IVIg replacement therapy (*SI Appendix*, Table S4). HSCT restored the ability of P1 to mount specific Ab responses against hepatitis B, rubella, and mumps. Similarly, naïve B cells isolated from P1 6 mo post-HSCT were able to produce IgM, IgG, and IgA in vitro at levels within the ranges observed for naïve B cells from healthy donors under all in vitro culture conditions tested (CD40L ± CpG/BCR or IL-21) (Fig. 7 *D*–*F*). Consistent with the kinetics of B cell immune reconstitution after HSCT, 3 y after transplantation, P1 had normal proportions of B cells, normal serum levels of IgG, IgA, and IgM, was off IVIg replacement therapy, and reported no B cell-related infections (*SI Appendix*, Table S4, and case report in *SI Appendix*). Overall, these results suggest that there is a B cell-intrinsic requirement of RelB for the generation of class-switched memory B cells and Ab-secreting cells, accounting for the severe hypogammaglobulinemia and recurrent infections observed in P1 (pretransplantation) and P2 (*SI Appendix*, Tables S4 and S5).

## Discussion

We describe two adult patients with AR RelB deficiency manifesting as CID with early-onset severe bacterial, viral, and fungal diseases. P1 (Q72Tfs152 variant), P2 (E145K/P364L variants), and the other three patients tested (Y397* variant) had low TREC counts or low proportions of recent thymic emigrant cells, suggestive of impaired thymic function (7, 23) (*SI Appendix*, Tables S4 and S5). In addition, P1, P2, and the other seven reported RelB-deficient patients (Y397*, P364L, and Q135dup variants) had low frequencies of naïve CD4^+^ T cells (23–25). P2 and one previously reported patient (Y397* variant) had skewed T cell Vβ repertoires, suggestive of a peripheral expansion of some T cell clones (7, 23) (*SI Appendix*, Tables S4 and S5). P1 and P2, like the three patients with the Y397* variant previously reported, had impaired but detectable antigen-specific T cell responses in vivo and in vitro (*Materials and Methods* and *SI Appendix*, Table S6) (7, 23). The B cell compartment was also affected, with low levels of memory B cells in P1, an almost total absence of class-switched memory B cells ex vivo and impaired B cell differentiation into IgM- and class-switched Ab-secreting cells in vitro in P1, and very low total B cell counts in P2. P1, P2, and the three siblings homozygous for the Y397* *RELB* variant presented with hypogammaglobulinemia and recurrent bacterial respiratory tract infections, and P2 (P1 was not tested) also displayed impaired T cell-dependent Ab responses. These findings are consistent with those for other inborn errors of the alternative NF-κB pathway, which impair B cell counts and Ig production over time (6, 8, 25). Together, these clinical and cellular phenotypes are strongly suggestive of a profound combined T- and B cell immunodeficiency in patients with partial or complete forms of AR RelB deficiency. Strikingly, the immunological and infectious phenotypes appear to be robust in these nine patients from five kindreds of different ancestries (Turkish, Irish, Iranian, Chinese, Scottish, French, and German) (7, 23, 24) (*SI Appendix*, Table S7).

Both P1 and P2 suffered from CMC, as reported in five of the seven patients with AR NIK deficiency and one patient with AR partial IKK-α deficiency, but not in patients with AD inborn errors of NF-κB2 (6, 9, 18, 19, 40, 46, 47). CMC has been associated with impaired IL-17A/F-mediated immunity (40). Consistently, P1 and P2 had low proportions of T_h_17 cells ex vivo, and P1 displayed low levels of IL-17A and IL-17F production by memory CD4^+^ T cells (P2 was not tested). This paucity of T_h_17 cells probably explains the CMC reported in P1 and P2 (40). By contrast, circulating T_h_17 cell levels were normal in patients with AD inborn errors of NF-κB2 (6). In addition to CMC, P1 also suffered from invasive fungal diseases and cryptococcal meningitis (*SI Appendix*, Table S4) (48). No auto-Abs neutralizing GM-CSF resembling those described in adult patients with disseminated disease due to *Cryptococcus* spp. were found (49). However, the promoter of the human *CSF2* gene, encoding GM-CSF, contains a κB motif. Furthermore, mouse RelB can bind to the *CSF2* promoter to induce NIK-dependent GM-CSF production and mouse *Relb-*KO T cells have impaired GM-CSF production (50–52). Impaired T cell-intrinsic RelB-dependent GM-CSF production may, therefore, have contributed to the susceptibility to *Cryptococcus* disease in P1.

P2 has suffered from severe viral diseases, including severe varicella pneumonia at the age of 3 y, recalcitrant common warts, and epidermodysplasia verruciformis since the age of 12 y, and he eventually died from PML at the age of 34 y. HPV lesions and PML may be related to the low CD4^+^ T cell counts found in P2 (53–56). The severe varicella may have been due to the neutralizing auto-Abs against type I IFNs detected in the plasma of P2 as an adult, as shown in patients with AD p52^LOF^/IκBδ^GOF^, who do not have CID (6), and in other patients with such auto-Abs in other contexts (6, 57–60). By contrast, P1 reported no severe viral infection associated with neutralizing auto-Abs against type I IFNs (detected at the age of 19 y, 5 y after HSCT, but not before HSCT, at the age of 14 y). Consistent with the critical mTEC-intrinsic role of p52/RelB heterodimers in establishing tolerance to type I IFNs, HSCT did not prevent the development of thymic stromal cell-dependent autoimmunity in P1, who developed auto-Abs against type I IFNs after transplantation. Her thymus was also hypoplastic, as reported in another AR *RELB*-deficient patient (Q135dup variant) and in *Relb*-KO mice (24, 61–63). The autoimmune phenotype observed in P1 and P2, restricted to the development of auto-Abs against type I IFNs, sharply contrasts with that of *Relb*-KO mice, which die from overwhelming T lymphocyte-dependent multiple-organ inflammation at the age of 2 to 6 wk (64–66). By contrast, the liver, lung, and skin-related autoimmunity reported in two patients with AR partial RelB deficiency (25) closely mimics the T cell infiltration of organs in mice with a conditional *Relb*-KO in mTECs (67), providing additional evidence for the mTEC-intrinsic essential role of human RelB in central tolerance. HSCT corrected the infectious susceptibility associated with T and B cells in the four patients with AR complete RelB deficiency who underwent transplantation. It would therefore be interesting to determine whether this procedure can also correct organ-related autoimmunity in patients with AR partial RelB deficiency.

All nine patients with AR RelB deficiency display CID, like patients with AR NIK or IKK-α deficiency, whereas patients with AD inborn errors of NF-κB2 display PAD (7–9, 15, 18, 19, 23, 24). Ectodermal dysplasia with nail dystrophy, sparse hair, and alopecia, as described in a third of patients with AD p52^LOF^/IκBδ^GOF^ and in all four patients with AR partial IKK-α deficiency, was not reported in the seven NIK-deficient patients, and subtle manifestations were found in only one of the nine patients with AR RelB deficiency (P2, sparse body hair during childhood) (8, 15, 18, 19). Some of the clinical and immunological differences between these patients could be explained by the difference in the IκBδ activity in primary fibroblasts after activation of the alternative NF-κB pathway. Indeed, an accumulation of unprocessed p100, conferring IκBδ^GOF^ activity, was found in the cells of patients with inborn errors affecting the processing of p100 (AR NIK deficiencies and AD p52^LOF^/IκBδ^GOF^), whereas p100 levels were low in the cells of patients with AR RelB deficiency, due to impaired RelB-dependent de novo transcription of *NFKB2*.

The experiments of Nature described here demonstrate that an absence of human RelB in hematopoietic cells severely disrupts the alternative NF-κB pathway, thereby impairing the T- and B-lymphoid branches of adaptive immunity at multiple levels, manifesting as weakened host defense against various infectious agents. They also reveal the essential mTEC-intrinsic role of RelB for T cell tolerance to type I IFNs in the human thymus. The correction of susceptibility to bacterial and fungal infections after successful HSCT confirms the presence of leukocyte-intrinsic defects in the absence of human RelB. However, these patients may remain at high risk of developing severe viral disease because of the persistence of auto-Abs against type I IFNs (68–70). These observations highlight the importance of evaluating changes in the levels of these auto-Abs over time. They also suggest that therapeutic options for correcting the thymic stromal defect in these patients, such as thymic implantation or stem-cell-based TEC regeneration, are worth considering (71). These findings also stress the importance of an in-depth characterization of the *RELB* mutant alleles, as four patients had complete forms and five patients had partial forms of AR RelB deficiency. This is clinically relevant, as patients with a partial defect seem to have a milder course of disease, although patients with both forms had CID with auto-Abs against type I IFNs.

## Materials and Methods

### Healthy Subjects.

The healthy subjects were volunteer blood donors of European, Australian, and Turkish origin.

### Blood Collection.

Blood samples were collected from all patients and relatives after written informed consent had been obtained. Blood samples were drawn as part of the diagnostic procedure. The study was approved by the institutional review boards of INSERM, Rockefeller University and Necker Hospital for Sick Children. Heparin-treated blood samples were obtained from healthy relatives of the patients. All these procedures were conducted in accordance with the 1975 Declaration of Helsinki, as revised in 2013. Blood was collected from P1 and P2 during periods free from immunosuppressive treatments.

### Molecular Genetics.

Genomic DNA was isolated from whole blood by a phenol/chloroform extraction method. *RELB* gDNA was amplified with specific primers (PCR amplification conditions and primer sequences are available upon request). PCR products were analyzed by electrophoresis in 1% agarose gels, sequenced with the Big Dye Terminator cycle sequencing kit (Applied Biosystems, Foster City, CA), and analyzed on an ABI Prism 3700 (Applied Biosystems, Foster City, CA).

### Whole-Exome Sequencing.

Genomic DNA (3 µg) extracted from the peripheral blood cells of P1 was sheared with a Covaris S2 Ultrasonicator (Covaris). An adaptor-ligated library was prepared with the Paired-End Sample Prep kit V1 (Illumina). Exome capture was performed with the Sure Select Human All Exon kit (Agilent Technologies). Single-end sequencing was performed on an Illumina Genome Analyzer IIx (Illumina), generating 72-base reads.

### Transfection of HEK293T Cells and Expression of *RELB* Variants.

The pCMV6 vector encoding C-terminally Flag-tagged human RelB was purchased from Origene (Maryland, USA). The construct carrying the mutant allele was generated by direct mutagenesis with the CloneAmp HIFI PCR Premix (Takara, Japan). Cells were transfected for 24 h, after which RelB was detected by immunoblotting with an antibody directed against the C terminus of RelB (D7D7W, Cell Signaling) or against the N terminus of RelB (ab33917, Abcam), with an antibody against GAPDH used as a loading control (FL-335, Santa Cruz Biotechnology).

### Luciferase Reporter Activity.

NF-κB luciferase activity was assessed by transiently transfecting HEK 293T cells with 100 ng of an NF-κB–dependent firefly luciferase vector with five NF-κB binding sites (5′-GGGGACTTTCC-3′) in the promoter (Agilent Technologies), 20 ng of the pRL4.74 *Renilla* luciferase vector (Promega) as an internal control and an empty pCMV6 plasmid (EV), a pCMV6 plasmid encoding WT RELB, P1’s variant (c.C212dup, p.Q72Tfs*152), P2’s variant (c.433G>A/c.1091C>T, p.E145K/p.P364L), the previously reported *RELB* variants (c.1191C>A, p.Y397*, and c. c.400_c.401insAGC/p.Q135dup) or the two gnomAD missense (c.1249 G > A, p.D417N, and c.1459 G > T, p.D487Y) variants of RelB (50 ng/well) ± a pCMV6-*NFKB2*-DDK WT plasmid (50 ng/well). The transfection mixture was prepared with 3% XtremeGene 9 DNA Transfection Reagent (Merck) and a serum-free medium, Optimem (Gibco). After incubation for 24 h, the cells were lysed in passive lysis buffer, and luciferase activities were measured in the Dual-Luciferase Reporter Assay (Promega).

## Supplementary Material

Appendix 01 (PDF)

Appendix 02 (PDF)

Dataset S01 (XLSX)

## Data Availability

All study data are included in the article and/or *SI Appendix*.
