# Supplementary material for: Inherited human RelB deficiency impairs innate and adaptive immunity to infection
Source: Proc Natl Acad Sci U S A. 2024 Sep 4;121(37):e2321794121. doi: 10.1073/pnas.2321794121 (PMC11406260; doi:10.1073/pnas.2321794121)
Supplement: Supplementary file 1 — Appendix 01 (PDF) [file pnas.2321794121.sapp1.pdf]

PNAS

Supplementary Information for

Inherited human RelB deficiency impairs innate and adaptive immunity to infection

Tom Le Voyer[a,b,c#], Majistor Raj Luxman Maglorius Renkilaraj[a,b,#], Kunihiko Moriya[a,b,#], Malena Pérez Lorenzo[a,b,¶], Tina Nguyen[d,e,¶], Liwei Gao[a,b,¶], Tamar Rubin[f,&], Axel Cederholm[g,&], Masato Ogishi[h,&], Carlos A. Arango-Franco[a,b,i], Vivien Béziat[a,b,h], Romain Lévy[a,b,h], Mélanie Migaud[a,b], Franck Rapaport[h], Yuval Itan[j], Elissa K Deenick[d,e], Irene Cortese[k], Andrea Lisco[l], Kaan Boztug[m,n,o], Laurent Abel[a,b,h], Stéphanie Boisson-Dupuis[a,b,h], Bertrand Boisson[a,b,h], Patrick Frosk[p], Cindy S Ma[d,e], Nils Landegren[g], Fatih Celmeli[q], Jean-Laurent Casanova[a,b,h,r,s,§,*], Stuart G Tangye [d,e,§], and Anne Puel[a,b,h,§,*]

#, ¶, &, § Equal contributions
* Correspondence: anne.puel@inserm.fr, casanova@rockefeller.edu

This PDF file includes:

SI Materials and Methods
Figures S1 to S8
Tables S1 to S7

SI Materials and Methods

Case report for P1

A 13-year-old female patient was referred to the pediatric immunology clinic for recurrent sinopulmonary infections beginning at the age of six years. The patient was born at full term to consanguineous parents, after a normal pregnancy. The delivery was uneventful. There was no family history of recurrent infections or immunological disorders. The patient had received all regular vaccinations, including those based on live vaccines (BCG and polio), without complications. At six years of age, she was diagnosed with oral candidiasis, otitis and recurrent respiratory tract infections leading to repeated hospital admissions. The patient had had multiple episodes of bronchopneumonia, resulting in bronchiectasis. At the age of 10 years, hypogammaglobulinemia was detected and the patient received short-term intravenous immunoglobulin treatment. During follow-up, at the age of 12 years, the patient presented with *Cryptococcus neoformans* meningitis complicated by cranial nerve palsy resulting in a loss of vision (26). The infectious phenotype of the patient, including chronic sinusitis, chronic otitis, oral candidiasis, and recurrent skin infections, was associated with a failure to thrive (weight and height below the third percentile for age) and mild splenomegaly. A marked macular eruption with hyperpigmentation was observed on the lower extremities. Laboratory test results were as follows: hemoglobin, 11 g/dL; platelets, 158000/mm^3; WBC, 9500/mm^3 and an absolute lymphocyte count of 3000 cells/mm^3. Mantoux tests for TB and serological tests for HIV were negative. Immunological testing revealed normal T-, B- and NK-lymphocyte counts, although the levels of recent thymic emigrants and TREC were below the lower limit of the normal range for age, and chest CT scans showed a thymus smaller than that in an age-matched healthy subject (**Table S4; Fig. S3A-B**). P1 had no pathologically enlarged lymph nodes during or independently of infectious disease. Serum Ig isotypes were evaluated before IVIg replacement therapy, at the age of 13 years: IgM levels were high, but

IgG and IgA levels were extremely low (**Table S4**). It was not possible to assess levels of antigen-specific antibodies whilst the patient was on IVIg replacement therapy. The patient had no autoimmune manifestations. She was negative for anti-dsDNA, ANA, RF, anti-cardiolipin, anti-M2, ANCA, anti-HTG, anti-TPO, anti-GAD, anti-insulin, and anti-islet antibodies (**Table S4**). The patient was diagnosed with CID. At the age of 14 years, she underwent HSCT with peripheral blood stem cells donated by a fully HLA-matched sibling, with a low-intensity conditioning regimen. The patient is currently in good health, with donor myeloid and T-cell chimerism levels > 90%, without GVHD. In particular, she has not presented any of the infections observed before transplantation, or any other severe infections. She suffered no serious complications from SARS-CoV-2 infection. No thymic hyperplasia was observed. The patient has been off IVIg replacement therapy since the age of 17 years. She has also been able to mount specific antibody responses to hepatitis B, rubella, and mumps vaccination. She has remained negative for anti-dsDNA, ANA, RF, anti-cardiolipin, anti-M2, ANCA, anti-HTG, anti-TPO, anti-GAD, anti-insulin, and anti-islet antibodies. However, auto-Abs against IFN-α2 and IFN-ω were detected five years after HSCT (100 pg/mL). The patient's IgG levels have normalized, and her IgM levels have decreased towards the normal range. No neutralizing auto-Abs against type I IFNs were detected in the plasma samples from the P1's parents (I.1 and I.2). No material from P1's siblings (II.2 and II.3) was available for testing.

Case report for P2

We recruited a second patient, a 33-year-old man of Irish, Scottish, French, and German descent. His mother had had chronic lymphocytic leukemia, neither the patient's non-consanguineous parents nor his brother presented any other unusual infectious, autoimmune or autoinflammatory manifestations. At the age of three months, P2 presented with bilateral chorioretinitis of unknown cause. He suffered from one episode of varicella pneumonia requiring mechanical ventilation at the age of three years. Since infancy, P2 has had

several episodes of bacterial peritonitis, pneumonia, otitis media, sinusitis, and oral and esophageal candidiasis. The patient reported a lower frequency of episodes of sinusitis and pneumonitis whilst on IVIg replacement therapy. Between the ages of five and six years, he suffered one episode of osteomyelitis. He reported having "sparse body hair" when assessed for delayed puberty during adolescence. A biopsy of warts on the hand and feet of P2 at the age of about 12 years revealed epidermodysplasia verruciformis. HPV typing revealed the presence of HPV 23. The warts progressed to the skin of the groin, anterior neck, trunk, arms, and temple areas. The lesions were refractory to multiple treatments and required debridement surgery. The patient developed an early-onset malabsorption syndrome associated with chronic asymmetric lymphedema of both legs from birth onwards and had suffered from diarrhea and failure to thrive since infancy. Gastrointestinal (GI) endoscopy and biopsies provided no evidence of lymphangiectasia. Alpha-one antitrypsin clearance studies did not detect protein-losing enteropathy in this patient, so the etiology of his early GI issues remains unclear. The patient also developed cirrhosis with cholestatic liver disease and portal hypertension. He had primary hypothyroidism, high TSH levels, and low gonadotropin and testosterone levels, but no detectable anti-TPO or anti-thyroglobulin auto-Abs. At the age of 22 years, P2 was diagnosed with EBV-negative diffuse large B-cell lymphoma (DLBCL) with meningeal and diffuse gastrointestinal involvement. Short-term treatment with IV dexamethasone was administered for DLBCL-related intracranial hypertension. The patient went into remission after chemotherapy with rituximab-cyclophosphamide, doxorubicin, vincristine, and prednisone (R-CHOP) and intrathecal cytarabine (Ara-C). At the age of 31 years, he was treated for JC virus encephalitis. He developed alopecia, which worsened after chemotherapy, and suffered from chronic lymphopenia. A comprehensive immunological analysis at the age of 17 years showed severe lymphopenia with low $CD4^+$ T-cell counts. The lymphocyte responses of this patient to pokeweed mitogen, phytohemagglutinin, concanavalin A, and *Candida* antigen ranged from normal to 20 to 30% the levels in normal healthy subjects, with low levels of proliferation in response

to VZV. TCR Vβ repertoire analysis showed an overrepresentation of TCR Vβ21.3, with parallel decreases in the levels of the other TCR Vβ families. P2 had low levels of $CD56^+$ natural killer cells, associated with a profound decrease in the function of these cells (in chromium release assays). P2 had 56 B cells/µL of blood at the age of 22 years, before rituximab infusion. B cells remained almost undetectable after the rituximab infusions, with fewer than 5 circulating $CD19^+$ cells/mm^3 at the age of 32 years. The patient responded poorly to immunization with several vaccines (poor response to mumps and varicella, despite prior vaccination against measles mumps and rubella, and despite having had chicken pox and shingles; anti-tetanus titers were 0.29 IU/mL and anti-diphtheria and anti-mumps antibody titers were below the limit of detection). P2 had hypogammaglobulinemia requiring IVIg replacement therapy from the age of 18 years. On clinical laboratory screening, P2 tested positive for anti-dsDNA autoantibodies, but negative for ANA and RF (**Table S5**). IgM levels were normal to high, IgA levels were low to normal and no circulating IgE antibodies were detected. P2 received trimethoprim/sulfamethoxazole prophylaxis continuously from the age of 22 years. He died from PML at the age of 34 years.

Cell purification and culture of the patients' cells

Human peripheral blood mononuclear cells (PBMCs) were isolated by Ficoll-Hypaque density centrifugation (Amersham Pharmacia Biotech, Sweden) from whole blood. Primary human fibroblasts obtained from skin biopsy specimens, and SV40 fibroblasts obtained after the immortalization of primary fibroblasts with SV-40T antigen (SV40-immortalized fibroblasts or SV40-F) were cultured in DMEM (Gibco BRL, Invitrogen, USA) supplemented with 10% fetal bovine serum (FBS) (Gibco BRL, Invitrogen, USA). All cells were grown at 37°C, under an atmosphere containing 5% CO_2.

Generation of EBV-B cells with *RELB* variants

PBMCs were isolated by Ficoll-Hypaque density centrifugation (Amersham-Pharmacia-Biotech) from whole-blood samples obtained from healthy volunteers or patients. EBV-immortalized lymphoblastoid cell lines were cultured in RPMI medium supplemented with 10% FBS.

Full-length RT-PCR for *RELB* and quantitative PCR analysis

Total RNA was extracted (Qiagen, France) and used as a template for cDNA synthesis with the High-Capacity cDNA Reverse Transcription Kit (Applied Biosystems). The cDNA for the full-length transcript was amplified with the following primers: forward primer: GTGCATGCTTCGGTCTGGGC, reverse primer: CTACGTGGCTTCAGGCCCC. Real-time quantitative PCR (qPCR) was performed with the Biosystems Assays-on-Demand probes/primers for TaqMan for *RELB* (Hs00232399_m1, Thermo Fisher Scientific), *NFKB2* (Hs00174517_m1, Thermo Fisher Scientific), *IL6* (Hs00985639_m1, Thermo Fisher Scientific) or *CSF2* (Hs00929873, Thermo Fisher Scientific). *GAPDH* (00402869, Thermo Fisher Scientific) was used for normalization. For RT-qPCR on primary leukocyte and myeloid subsets, cells from freshly isolated PBMCs were negatively (NK, naïve and memory $CD4^+$ and $CD8^+$ T cells) or positively ($CD19^+$, $CD14^+$, pDC, mDC) sorted by magnetic separation (Miltenyi Biotec). The results are expressed according to the $2^{-\Delta\Delta Ct}$ method, as described by the manufacturer.

Western blot

Western blots were performed as previously described (6). Whole-cell lysates from HEK293T cells, primary fibroblasts or SV40-F were prepared in RIPA buffer (50 mM Tris-HCl, pH 7.5, 150 mM NaCl, 1% NP40, 0.5% sodium deoxycholate, and 0.1% SDS) supplemented with Complete Protease Inhibitor Cocktail (Roche). Equal amounts of protein, according to a Bradford protein assay (Bio-Rad, Hercules, California, USA), were resolved by SDS-PAGE in a Criterion™ TGX™ 10% precast gel (Bio-Rad) and the resulting

bands were transferred to a polyvinylidene fluoride membrane (Millipore). All blots were incubated overnight with primary antibodies and developed with the Pierce ECL Western Blotting Substrate (Thermo Fisher Scientific). The antibodies used in this study included antibodies against p100/p52 (Cat#4882; Cell Signaling Technology, dilution 1/1000), p105/p50 (N-terminus; Cat#3035; Cell Signaling Technology, dilution 1/1000), p65 (Cat#sc-372; Santa Cruz Biotechnology, dilution 1/1000), C-terminus of RelB (Cat#sc-48366; Santa Cruz Biotechnology, dilution 1/800), N-terminus of RelB (Cat#ab33917, Abcam), c-Rel (Cat#sc-6955; Santa Cruz Biotechnology, dilution 1/1000), IKK-α (B-8, Santa Cruz Biotechnology), IκB-α (C-21, Santa Cruz Biotechnology), and the following secondary antibodies: Amersham ECL mouse IgG, HRP-linked whole antibody (from sheep; Cat#NA931; GE Healthcare Life Sciences) and Amersham ECL rabbit IgG, HRP-linked whole antibody (from donkey; Cat#NA934; GE Healthcare Life Sciences). Antibodies against GAPDH (FL-335, Santa Cruz Biotechnology), tubulin (B-7, Santa Cruz Biotechnology) and laminin A/C (H-110, Santa Cruz Biotechnology) were used as loading controls. The appropriate HRP-conjugated secondary antibodies were incubated with the membrane for the detection of antibody binding with the ChemiDoc MP system (Bio-Rad). Quantification was performed with Image Labo software (Biorad).

Confocal microscopy

Confocal microscopy was performed as previously described (6). HeLa cells were plated on chambered coverslips (Cat#80826, iBidi) and were left untransfected or were transiently transfected with a plasmid encoding p100, RelB and/or NIK and/or an empty pCMV6 vector for 48 hours. Primary fibroblasts or SV40-F were plated on chamber coverslips and left unstimulated or were stimulated with 100 ng/mL Lt or 100 ng/mL TWEAK for 48 hours. The cells were fixed in 4% formaldehyde in phosphate-buffered saline (PBS), pH 7.4. Cells were incubated overnight at 4°C with anti-p105/p50 (Cat#sc-3035; Santa Cruz

Biotechnology, dilution 1/1000), anti-p65 (Cat#sc-372; Santa Cruz Biotechnology, dilution 1/1000), anti-p100/p52 (Cat#4882; Cell Signaling Technology, dilution 1/1000), or anti-RelB (Cat#sc-48366; Santa Cruz Biotechnology, dilution 1/800) primary antibodies. The cells were washed three times with 1X PBS and incubated with secondary antibodies for 1 h at room temperature (goat anti-mouse IgG Alexa Fluor 488 (Cat#A-11029, dilution 1/250); goat anti-rabbit IgG Alexa Fluor 633 (Cat#A-11037, dilution 1/250) before mounting in Prolong-gold and visualization by confocal microscopy (×63 or x40 oil immersion lens).

Simulation of primary fibroblasts or SV40-F and qPCR

Primary skin fibroblasts or SV40-F were grown to about 80% confluence. Cells were harvested by trypsin treatment and used to reseed new plates at a density of 1.6 x 10^5 cells per well (six-well format). Primary fibroblasts were grown in 2 mL of 10% SVF in DMEM and SV40-F were incubated in 2 mL of 2% FBS in DMEM per well, in a six-well plate. Cells were either left unstimulated or were stimulated for 24 h with various ligands: 10 ng/mL IL-1β (R&D Systems), 20 ng/mL TNF (R&D Systems), 10 µg/mL 2,3-bis(palmitoyl oxy)propyl (PAM)-2 (TLR2 agonist), 10 µg/mL PAM-3 (TLR3 agonist), 1 µg/mL FSL-1 (TLR2/6 agonist), 10 µg/mL lipoteichoic acid from *S. aureus* (LTA-SA) (TLR2 agonist), 1 µg/mL MPLA (TLR4 agonist) (all from InvivoGen), 1 µg/mL lipopolysaccharides (rough strains) from *Salmonella enterica* serotype Minnesota Re 595 (LPS; Sigma), or 25 µg/mL poly(I:C) (GE Healthcare), 100 ng/mL lymphotoxin α1β2 (Lt, R&D Systems), or 100 ng/mL TWEAK (R&D Systems) in medium. Cells were harvested and lysed for the assessment of target gene expression by RT-qPCR.

Lentiviral transduction of SV40 fibroblasts

Retroviral vectors expressing the WT *RELB*, a variant from one of our patients, c.212 dup, the previously reported *RELB* mutant, c.1191 C>A, or one of the two missense gnomAD variants, c.1249 G>A or c.1459

G>T, were generated by inserting the *RELB* sequence from the corresponding pCM6-*RELB*-DDK plasmid into pTRIP-SFFV-ΔNGFR-2A. The plasmids were used to transfect HEK293T cells along with pCMV-VSV-G (Addgene Plasmid #8454), psPAX2 (Addgene Plasmid #12260) and pHXB2 (NIH-AIDS Reagent Program, 1069), with Opti-MEM (Gibco) and X-tremeGENE 9 (Roche), according to the manufacturers' instructions. Six hours after transfection, the medium was replaced, and 24 hours later, the supernatants were collected, filtered through 25 mm Acrodisc Syringe Filters with a 0.2 µm-mesh Supor Membrane (Pall Corporation), and subjected to precipitation with the Retro-X concentrator (Clontech), according to the manufacturer's instructions. One million SV40 fibroblasts were mixed with lentivirus-containing supernatant in a total volume of 2 mL. After 24 h of incubation, 200 µL of FCS was added and the cells were incubated for a further four days. Transduced cells were purified by positive MACS with an anti-NGFR-biotin antibody and an anti-biotin antibody conjugated with magnetic beads (Miltenyi Biotec), according to the manufacturer's protocol. The levels of *NFKB2* and *RELB* expression were assessed by RT-qPCR.

Luciferase neutralization assay for auto-Abs against type I IFNs

The blocking activity of anti-IFN-α2 and anti-IFN-ω auto-Abs was determined with a reporter luciferase assay. Briefly, HEK293T cells were transfected with a plasmid containing the firefly luciferase gene under the control of the human *ISRE* promoter in the pGL4.45 backbone, and a plasmid constitutively expressing *Renilla* luciferase for normalization (pRL-SV40). Cells were transfected in the presence of the X-tremeGene 9 transfection reagent (Sigma Aldrich, Cat#6365779001) for 24 hours. Cells in Dulbecco's modified Eagle medium (DMEM, Thermo Fisher Scientific) supplemented with 2% fetal calf serum (FCS) and 10% healthy donor or patient serum/plasma were either left unstimulated or were stimulated with IFN-α2 (Miltenyi Biotec, Cat#130-108-984) or IFN-ω (Merck, Cat#SRP3061) at 10 ng/mL or 100 pg/mL, or

with IFN-β (Miltenyi Biotech, Cat#130-107-888) at 10 ng/mL, or 1 ng/mL, or with one of the 13 IFN-α subtypes for 16 hours at 37°C. Each sample was tested once for each cytokine and dose, in at least two independent experiments. Finally, cells were lysed for 20 minutes at room temperature and luciferase levels were measured with the Dual-Luciferase® Reporter 1000 assay system (Promega, Cat#E1980), according to the manufacturer's protocol. Luminescence intensity was measured with a VICTOR X Multilabel Plate Reader (PerkinElmer Life Sciences, USA). Relative luciferase activity (RLA) was calculated by normalizing firefly luciferase activity against *Renilla* luciferase activity, and then normalizing against non-stimulated conditions. Samples were considered to be neutralizing if the luciferase activity signal, normalized against non-stimulated conditions, was below 5.

Protein microarray

Protein microarrays (HuProt, CDI Laboratories) were performed as previously described (6). Protein microarrays were incubated for 90 minutes in 5 mL of a blocking solution consisting of 2% bovine serum albumin and 0.05% Tween-20 in PBS. The arrays were then immersed overnight in 5 mL blocking solution per array with serum from either a blood donor or a patient at a 1:2,000 dilution. Each array then underwent five five-minute wash cycles with 5 mL PBS-T (PBS + 0.05% Tween-20). Alexa Fluor 647 goat anti-human IgG (Thermo Fisher Scientific, A-21445, RRID:AB_2535862) and Dylight 550 goat anti-GST (Columbia Biosciences, D9-1310) were diluted in blocking solution (at dilutions of 1:2,000 and 1:10,000, respectively). Each array was immersed in 5 mL of this mixture for 90 minutes. Five washes were then performed as described above. The incubations and washes were carried out on an orbital shaker, with aluminum foil used to block out light during steps involving fluorescent antibodies. Ultimately, each array was thoroughly rinsed three times with deionized water and centrifuged for approximately 30 seconds for drying purposes. Later the same day, an Innoscan 1100AL Fluorescence scanner (Innopsys) in tandem with Mapix v.9.1.0

was used to scan the arrays. The resulting images underwent analysis using the Jan 18-22 Huprot v4.0 Genepix Array List file, with either GenePix Pro v.5.1.0.19 or GenePix Pro 7. A normalization procedure was applied to address variations in signal intensity across experiments. The dataset also included data from additional healthy donors from independent protein array experiments. Signal intensities were derived from the scanned image by subtracting the local background. IgG-reactive proteins were identified as proteins with a fluorescence intensity $\log_2$[fold change] ≥ 1.5.

Phenotyping of innate lymphoid cells

PBMCs were stimulated with PMA (10 ng/mL; Sigma-Aldrich) plus ionomycin (1 µg/mL; Sigma Aldrich) in the presence of Golgi-Plug (BD) for 3 h. Cells were surface-stained with mAbs against CD3 (UCHT1, eBioscience), CD4 (OKT4, eBioscience), CD5 (UCHT2, eBioscience), CD14 (TUK4, Miltenyi Biotec), CD19 (LT19, Miltenyi Biotec), TCR-αβ (IP26, eBioscience), TCR-γδ (B1.1, eBioscience), CD7 (M-T701, BD), CD56 (B159, BD), CD127 (eBioRDR5, eBioscience), and CD117 (104D2, Biolegend). Cells were fixed, permeabilized with the Foxp3/TF Staining Buffer Set (eBioscience) and stained with intracellular mAbs against EOMES (WD1928, eBioscience), GATA3 (TWAJ, eBioscience), and IL-13 (JES10-5A2, BD). Samples were analyzed on an LSR Fortessa machine (BD) and analyzed with FlowJo software (Tree Star). ILCs were gated as $Lin^-CD7^+CD56^-CD127^+$ cells. Lineage staining (Lin) included staining for CD3, CD4, CD5, CD14, CD19, TCR-αβ and TCR-γδ. ILC2 ($GATA3^+IL\text{-}13^+$) and ILCP ($CD117^+$) were distinguished within the ILC gate. All gated ILCs were $EOMES^-$.

Flow cytometry for the cT_{fh} and NK cell subsets

Immunophenotyping was performed by flow cytometry, with mAbs against CCR4 (REA279, Miltenyi Biotec), CCR6 (REA190, Miltenyi Biotec), CCR7 (G043H7, Sony), CD1c (L161, Biolegend), CD3 (7D6,

Invitrogen; UCHT1, BD), CD4 (RPA-T4, BD), CD5 (53-7.3, eBioscience), CD7 (M-T701, BD), CD8 (RPA-T8, BD), CD11c (S-HCL-13, BD), CD14 (M5E2, BD), CD16 (VEP13, Miltenyi Biotec; 3G8, BD), CD19 (4G7, BD or LT19, Miltenyi Biotec), CD20 (LT20, Miltenyi Biotec; H1, BD), CD24 (ML 5; BD), CD25 (MA-251, BD), CD27 (O323, Sony; L128, BD), CD45RA (Hl100, BD; T6D11, Miltenyi Biotec), CD56 (B159, BD; NCAM16.2, BD), CD123 (6H6, Biolegend), CD141 (1A4, BD), CD161 (DX12, BD), CXCR3 (REA232, Miltenyi Biotec), CXCR5 (REA103, Miltenyi Biotec), FOXP3 (259D/C7, BD), HLA-DR (L243, Biolegend), IgA (IS11-8E10, Miltenyi Biotec), IgG (G18-145, BD), IgM (PJ2-22H3, Miltenyi Biotec), KIR2DL1/S1 (EB6, Beckman Coulter) KIR2DL2/S2/L3 (GL183, Beckman Coulter), KIR3DL1 (DX9, Biolegend), NKG2A (REA110, Miltenyi Biotec), TCR-iNKT/Vα24JαQ (6B11, BD), TCR-γδ (11F2, Miltenyi Biotec), and TCR-Vα7.2 (REA179, Miltenyi Biotec). Cells were also stained with the Aqua Dead Cell Stain Kit (Thermo Fisher Scientific). When required, after extracellular staining, cells were fixed and permeabilized with a fixation/permeabilization kit (eBioscience) for intracellular staining. Samples were analyzed with a Fortessa X20 (BD) or Gallios (Beckman Coulter) machine, depending on the experiment. Data were then analyzed with Flow-Jo 10.1r5 software.

Immunophenotyping of primary leukocytes by spectral flow cytometry

Thawed PBMCs (1.2×10^6 cells per panel) were stained with LIVE/DEAD Fixable Blue dye (Thermo Fisher Scientific) and blocked by incubation with FcR blocking reagent (Miltenyi Biotec) on ice for 15 minutes. The cells were washed, and surface-stained by incubation with the following reagents on ice for 30 minutes: Brilliant Stain Buffer Plus (BD, Cat: 566385), anti-γδTCR-BUV661 (11F2, BD), antiCXCR3-BV750 (1C6, BD), and anti-CCR4-BUV615 (1G1, BD) antibodies. Cells were then washed and surface-stained by incubation with the following reagents on ice for 30 minutes: anti-CD141-BB515 (1A4), anti-CD57-FITC (HNK-1), anti-Vδ2-PerCP (B6), anti-Vα7.2-PerCP-Cy5.5 (3C10, BioLegend), anti-Vδ1-PerCP-Vio700

(REA173, Miltenyi Biotec), anti-CD14-Spark Blue 550 (63D3), antiCD1c-Alexa Fluor 647 (L161), anti-CD66b-APC (G10F5), anti-CD38-APC-Fire 810 (HB-7), antiCD27-APC H7 (M-T271), anti-CD127-APC-R700 (HIL-7R-M21), anti-CD19 Spark NIR 685 (HIB19), anti-CD45RA-BUV395 (5H9), anti-CD16-BUV496 (3G8,), anti-CD11b-BUV563 (ICRF44, BD), anti-CD56-BUV737 (NCAM16.2), anti-CD8-BUV805 (SK1, BD), MR1 tetramer-BV421, anti-CD11c-BV480 (B-ly6), anti-CD45-BV510 (HI30), anti-CD33-BV570 (WM53), anti-iNKT/Vα24JαQ-BV605 (6B11), anti-CD161-BV650 (DX12, BD), anti-CCR6-BV711 (G034E3), anti-CCR7-BV785(G043H7), anti-CD3-Pacific Blue (SK7,), anti-CD20-Pacific Orange (HI47), anti-CD123-Super Bright 436 (6H6), anti-Vβ11-PE (REA559, Miltenyi Biotec), anti-CD24-PE-Alexa Fluor 610 (SN3), anti-CD25-PE-Alexa Fluor 700 (3G10), anti-CRTH2-Biotin (BM16, Thermo Fisher Scientific), anti-CD209-PE-Cy7 (9E9A8), anti-CD117-PE-Dazzle 594 (104D2), anti-HLA-DR-PE-Fire 810 (L243), and anti-CD4-cFluor 568 (SK3) antibodies. The cells were washed and further incubated with streptavidin-PE-Cy5 (BioLegend, 1:3000) on ice for 30 minutes. Cells were then washed, fixed with 1% paraformaldehyde (PFA)/PBS, washed again, and acquired with an Aurora cytometer (Cytek). Subsets were manually gated with FlowJo software and further analyzed in R.

T_h17 cells *ex vivo*

For the *ex vivo* evaluation of IL-17A-, IL-17F-, IL-22- and IFN-γ-producing T cells by flow cytometry, PBMCs were purified by centrifugation on a gradient (Ficoll-Paque PLUS; GE Healthcare) and dispensed into 48-well plates at a density of 3×10^6 cells/mL in RPMI supplemented with 10% FBS. They were incubated for 12 hours with 40 ng/mL PMA (Sigma-Aldrich) plus 10^{-5}M ionomycin (Sigma-Aldrich), or T-cell activation and expansion beads coated with antibodies directed against CD2, CD3 and CD28 (130-091-441; Miltenyi Biotec) in the presence of a secretion inhibitor (1 µL/mL GolgiPlug; BD). The cells were washed in cold PBS and surface-labeled by incubation with PE-Cy™7 mouse anti-human CD3 (SK7; BD

Biosciences), CD4-APC-Vio770, human (M-T321; Miltenyi Biotec), Brilliant Violet 421™ anti-mouse CD197 (CCR7) (G043H7; Biolegend), PE-CF594 mouse anti-human CD56 (B159; BD Biosciences) antibodies and LIVE/DEAD® Fixable Aqua Dead Cell (L34957; Thermo Fisher Scientific) in 2% FBS/2 mM EDTA in PBS for 20 minutes on ice. Cells were washed twice with 2% FBS/2 mM EDTA in PBS, fixed by incubation with 100 µL BD Cytofix for 30 minutes on ice, and washed twice with BD Cytoperm (Cytofix/Cytoperm Plus, fixation/permeabilization kit; BD Biosciences). Cells were then incubated for 1 h on ice with antibodies purchased from eBiosciences: anti-human IL-17A Alexa Fluor® 488 (eBio64DEC17), anti-human IL-17F PE (SHLR17), anti-human/mouse IL-22 APC (IL22JOP) and anti-human IFN gamma Alexa Fluor® 700 (4S.B3) antibodies. They were washed twice with Cytoperm and analyzed with a FACS Gallios cell sorter. For the *ex vivo* evaluation of IL-17A- and IL-22- producing T cells by ELISA, we used 250 µL whole blood diluted in RPMI (500 µL final volume) to seed 48-well plates. The cells were incubated with 40 ng/mL PMA and 10^{-5} M ionomycin. Supernatants were collected after 24 h of incubation, for ELISA (R&D Systems).

***In vitro* differentiation of effector memory CD4^{+} T cells**

CD4^{+} T cells were labeled with anti-CD4, anti-CD45RA, anti-CCR7, anti-CD127 and anti-CD25 antibodies. Tregs (CD4^{+}CD25^{hi}CD127^{lo}) were excluded and effector/memory (defined as CD45RA^{-}CCR7^{+}) CD4^{+} T cells were isolated (> 98% purity) with a FACSAria machine (BD Biosciences). Purified naïve or effector/memory CD4^{+} cells were labeled with CFSE and cultured with T-cell activation and expansion beads (anti-CD2/CD3/CD28 mAb-coated; Miltenyi Biotec) alone (T_h0), under T_h1 (50 ng/mL IL-12), T_h2 (100 U/mL IL-4), or T_h17 (2.5 ng/mL TGFβ, 20 ng/mL IL-1β, 50 ng/mL IL-6, 50 ng/mL IL-21, 100 ng/mL IL-23, and 50 ng/mL PGE2) polarizing conditions for five days. Cytokine expression was assessed with activated CD4^{+} T cells, which were restimulated with PMA (100 ng/mL) and ionomycin (750 ng/mL) for

six hours, with the addition of brefeldin A (10 µg/mL) after two hours. The cells were fixed in 2% formaldehyde (Sigma-Aldrich), and intracellular cytokine expression was assessed by FACS. Culture supernatants were also used for cytometric bead array assays (BD Biosciences) or ELISA (Peprotech), to assess the secretion of the cytokines indicated. Viability was assessed by FACS after Zombie dye fixation (Biolegend), which showed a mean proportion of 94% living cells for controls and 58% living cells for the subsets of $CD4^+$ cells from P1.

In vitro proliferation and activation of B cells

Naïve B cells were sorted from peripheral blood. They were cultured in the presence of CD40L (200 ng/mL; R&D Systems) alone or with CpG 2006 (1 µg/mL; Sigma) ± F(ab')2 fragments of goat anti–human IgM (Jackson Laboratories), BAFF (PeproTech) or IL-21 (50 ng/mL; Peprotech). Their proliferation rate (with CD40L ± IL-21) was assessed by CFSE dilution. After five days, the induction of surface-cell activation markers (HLA-DR, CD80, CD86, CD95, IL-21R) was evaluated (with CD40L ± CpG ± BCR or BAFF or IL-21). After seven days, the production of IgG, IgA and IgM was assessed by Ig heavy chain-specific ELISA on the culture supernatant. Viability was assessed by FACS after Zombie dye fixation (Biolegend).

Supplementary Figures

Fig. S1. Confirmation, by WES analysis and Sanger sequencing, of the *RELB* variants in the families of P1 and P2, and population genetics of *RELB*

(**A**) Whole-exome sequencing (WES). We hypothesized that a biallelic variant (possibly even a copy number variant) could account for the unusually severe immunological abnormalities of P1, who was born to consanguineous parents. Variants were filtered on the basis of allele frequencies and predicted impact at the protein level (exclusion of non-coding or synonymous variants), or on the basis of a high gene damage

score or low variant MSC score (56, 59). Variants also found in the homozygous state in other healthy family members were discarded. We also discarded variants of genes for which homozygous variants predicted to be loss-of-function (pLOF) were found in the gnomAD database. After the application of these filters, we were left with eight homozygous variants (**Table S1**) and 38 compound heterozygous variants for P1. No candidate compound heterozygous variants of genes known to underlie inborn errors of immunity (IEI) were identified. Only one variant present in the homozygous state, a variant of *RELB*, c.C212dup, predicted to be LOF due to a premature stop codon generated by a frameshift, was considered a good candidate. The same filtering process was applied for P2, leading to the identification of two variants of *RELB*: C.433G>A and c.1091C>T. (**B**) Chromatograms showing the c.C212dup *RELB* variant in PBMC-derived DNA from P1 and relatives (black arrow), and comparison with a healthy (WT) donor. (**C**) Amino-acid residue conservation between species for the E145 and P364 positions of the RelB protein. (**D**) Gene-level negative selection. *RELB* is under strong negative selection, like other genes with mutations underlying AR or AD IEI, as determined by CoNeS (62). (**E**) Distribution of the *f* parameter for 18,968 human genes. A low *f* parameter indicates that the gene is under strong purifying selection (94). (**F**) HEK 293T cells were transiently transfected with an empty pCMV6 plasmid (empty vector, EV), or with pCMV6 plasmids containing the WT, c.C212dup (p.Q72fs, P1), c.1191 C>A (p.Y397*), c.433G>A (p.E145K, P2), c.1091C>T (p.P364L, P2), c.1249 G>A (p.D417N), c.1459 G>T (p.D487Y), or c.400_c.401insAGC (p.Q135dup) *RELB* cDNA. The RNA extracted from these cells was subjected to RT-qPCR for total *RELB*. Data are displayed as $2^{-\Delta Ct}$ values relative to *GUSB*. The results of three independent experiments are shown.

Fig. S2. RelB mRNA and protein levels and rescue of defective non-canonical NF-κB signaling in P2 fibroblasts

(**A**) RNAs extracted from EBV-B cells from healthy controls (C), P1's heterozygous mother (Mo), and P1 were subjected to RT-qPCR for total *RELB*. Data are expressed as $2^{-\Delta Ct}$ values relative to the mean expression of controls, after normalization against *GAPDH* (endogenous control) expression (ΔCt). (**B**) RNA extracted from EBV-B cells from two healthy donors (C1, C2) and P1 was subjected to RT-PCR to amplify the full-length *RELB* cDNA. Human *ACTB* (encoding β-actin) was used as an endogenous gene for comparison. (**C**) Immunoblot analysis of whole-cell lysates of EBV-B cells from four independent healthy subjects (C1-C4), P1, and P1's heterozygous mother (Mo) for the non-canonical NF-κB pathway components RelB, p100, and p52, with GAPDH used as a loading control. Quantification of p100, p52, and the p100/p52 ratio is shown on the right. The results shown are representative of three experiments. (**D**) Immunoblot analysis of RelB levels in SV40-F from healthy controls (C1-C3), P1, P1's heterozygous father (Fa), and a patient with AR complete NIK deficiency ($NIK^{-/-}$), with a rabbit mAb against the C-terminal residues of RelB. GAPDH immunoblotting was used as a loading control. The results shown are representative of three independent experiments. (**E**) RNA extracted from three different healthy subjects (Ctls), P1, P2 and a $NIK^{-/-}$ patient were stimulated with TWEAK for 24 h and subjected to RT-qPCR for *NFKB2* (upper panel) or *VCAM1* (lower panel). (**F**) SV40-F from a control (C1), the two RelB-deficient patients (P1 and P2, in red), a $NIK^{-/-}$ patient, and a patient with a $p52^{LOF}/I\kappa B\delta^{GOF}$ variant were left unstimulated or were stimulated with TWEAK for 48 h. NF-κB2 and RelB were detected with a mouse anti-p100 antibody (N-terminal) and with a rabbit anti-RelB antibody (C-terminal), respectively. Nuclei were labeled with DAPI. (**G**) SV40-F from P2 were stably transduced with an empty vector (EV) or WT *RELB* cDNA. The RNA extracted from these cells was subjected to RT-qPCR for total *RELB*. Data are displayed as $2^{-\Delta Ct}$ relative to EV-transduced cells, after normalization against *GUSB* (endogenous control) expression (ΔCt). The result of one representative experiment is shown. (**H**) SV40-F from P2 was stably transduced with an empty vector (EV) or WT *RELB* cDNA. The cells were either left unstimulated (NS,

blue bars) or were stimulated for 24 hours with Lt (black bars). The RNA extracted from these cells was subjected to RT-qPCR for total *NFKB2*. Data are displayed as $2^{-\Delta Ct}$ values relative to unstimulated EV-transduced cells, after normalization against *GUSB* (endogenous control) expression (ΔCt). The result of one independent experiment is shown. (**I**) SV40-F from a healthy control (C) and a NIK-deficient patient (NIK) were either not transduced (NT), or were stably transduced with an empty vector (EV) or the WT *MAP3K14* cDNA. The RNA extracted from these cells was subjected to RT-qPCR for total *NFKB2*. Data are displayed as $2^{-\Delta Ct}$ relative to EV-transduced cells, after normalization against *GAPDH* (endogenous control) expression (ΔCt). The results of one experiment are shown.

Fig. S3. Normal activation of the canonical NF-κB pathway in P1's fibroblasts

(**A**) Time course of activation of the canonical NF-κB pathway in TNF- (left) or IL-1β-(right) stimulated SV40-F from one healthy donor (C), P1, and NEMO-deficient SV40-F ($NEMO^{Y/-}$), as assessed by immunoblotting for IκB-α, RelB, NF-κB1 (p105/p50), RelA, and c-Rel, with GAPDH as a loading control. (**B**) IL-6 production by SV40-F from two healthy subjects (C), P1, and an IRAK4-deficient patient ($IRAK4^{-/-}$), left non-stimulated (NS), or stimulated with PAM-2 (10 µg/mL), PAM-3 (10 µg/mL), FSL-1 (1 µg/mL), LTA (10 µg/mL), LPS (10 µg/mL), MPLA (1 µg/mL), poly(I:C) (25 µg/mL), TNF (20 ng/mL), or IL-1β (10 ng/mL) for 24 hours. The values shown (means ± SEM) were obtained in three independent experiments.

Fig. S4. Auto-Abs against type I IFNs in P1 and P2

Heatmap showing the auto-Abs displaying the strongest enrichment in P1 and P2, as determined by HuProt, relative to individuals with APS-1 (*n*=15) and healthy controls (HC, *n*=25). Data are shown as log_2-fold changes normalized against the mean of HC.

Fig. S5. Immunophenotyping: development of innate and innate-like cells, dendritic cells, cTfh and Treg in P1 and P2

Immunophenotyping of PBMCs from healthy subjects (C, *n*=4-54), P1 (in red) and P2 (in blue). (**A**) Frequencies of NK cells ($CD3^{-}CD56^{+}$), iNKT cells ($CD3^{+}TCR^{-}V\alpha24J\alpha Q^{+}$), MAIT cells ($CD3^{+}CD161^{+}TCR\text{-}v\alpha7.2^{+}$), γδ T cells ($CD3^{+}TCR\text{-}\gamma\delta^{+}$), cDC1 ($Lin^{-}HLA\text{-}DR^{+}CD11c^{+}CD1c^{+}CD141^{-}$), cDC2 ($Lin^{-}HLA\text{-}DR^{+}CD11c^{+}CD1c^{-}CD141^{+}$), mDCs ($Lin^{-}HLA\text{-}DR^{+}CD11c^{+}CD123^{-}$), pDCs ($Lin^{-}HLA\text{-}DR^{+}CD11c^{-}CD123^{+}$), total ILCs ($Lin^{-}CD7^{+}CD56^{-}CD127^{+}CD45^{+}$), ILCPs ($CD117^{+}CD45^{+}$), and ILC2 ($GATA3^{+}IL\text{-}13^{+}CD45^{+}$) among the PBMCs of healthy subjects, P1 before HSCT, and P2. Frequency of $CD56^{bright}$ cells within the NK cell compartment of healthy subjects, P1, and P2, and terminal differentiation profile of the $CD56^{dim}$ compartment of healthy subjects, and P1. (**B**) Frequency of the cT_{fh} ($CXCR5^{+}$) cell subset within the $CD4^{+}$ memory compartment of healthy subjects (*n* = 13), and P2. (**C**) Absolute numbers of T_{reg} ($CD3^{+}CD4^{+}CD25^{hi}FoxP3^{+}$) and cT_{fh} ($CXCR5^{+}$) cells in healthy subjects (*n* = 5-13) and P2.

Fig. S6. Expression of activation markers in P1's naïve $CD4^{+}$ T cells under T_h0 or T_h1 polarizing conditions

Naïve T cells from six healthy donors (C) and P1 before HSCT were stimulated under T_h0 or T_h1 polarizing conditions, with medium only (Med.), or were incubated without stimulation (D0) for three days. Flow cytometry was then performed to assess the following cell-surface activation markers: CXCR5, CD40L, PD-1, CD25, CD69, and ICOS.

Fig. S7: RelB deficiency compromises memory $CD4^{+}$ T-cell cytokine production

(**A**) Proportions of cytokine-producing memory $CD4^+$ T cells, measured by flow cytometry, and (**B**) cytokine production, measured by ELISA, for IL-2, TNF, IFN-γ, IL-4, IL-5, IL-9, IL-10, IL-13, IL-21, IL-17A, IL-17F, and IL-22, after four days of culture under T_h0, T_h1, or T_h17 conditions, for cells from healthy donors (*n*=9), and from P1 before (pre-) and after (post-) HSCT.

Fig. S8: RelB deficiency impairs peripheral B-cell function in P1

(**A**) Rate of proliferation of naïve B cells from five healthy donors (black dots) and from P1 before HSCT (red dots), assessed by CFSE dilution after incubation with CD40L, CD40L + CpG, CD40L + CpG + BCR, CD40L + CpG + BAFF, or CD40L + IL-21. (**B**) Cell-surface marker expression for the same subjects, assessed by flow cytometry after incubation with CD40L ± IL-21 for 5 days, medium only (Med.), or on D0 without stimulation, for HLA-DR, CD80, CD86, CD95 and IL-21R.
