# Supplementary material for: Inherited human RelB deficiency impairs innate and adaptive immunity to infection
Source: Proc Natl Acad Sci U S A. 2024 Sep 4;121(37):e2321794121. doi: 10.1073/pnas.2321794121 (PMC11406260; doi:10.1073/pnas.2321794121)

Supplementary Figure 1

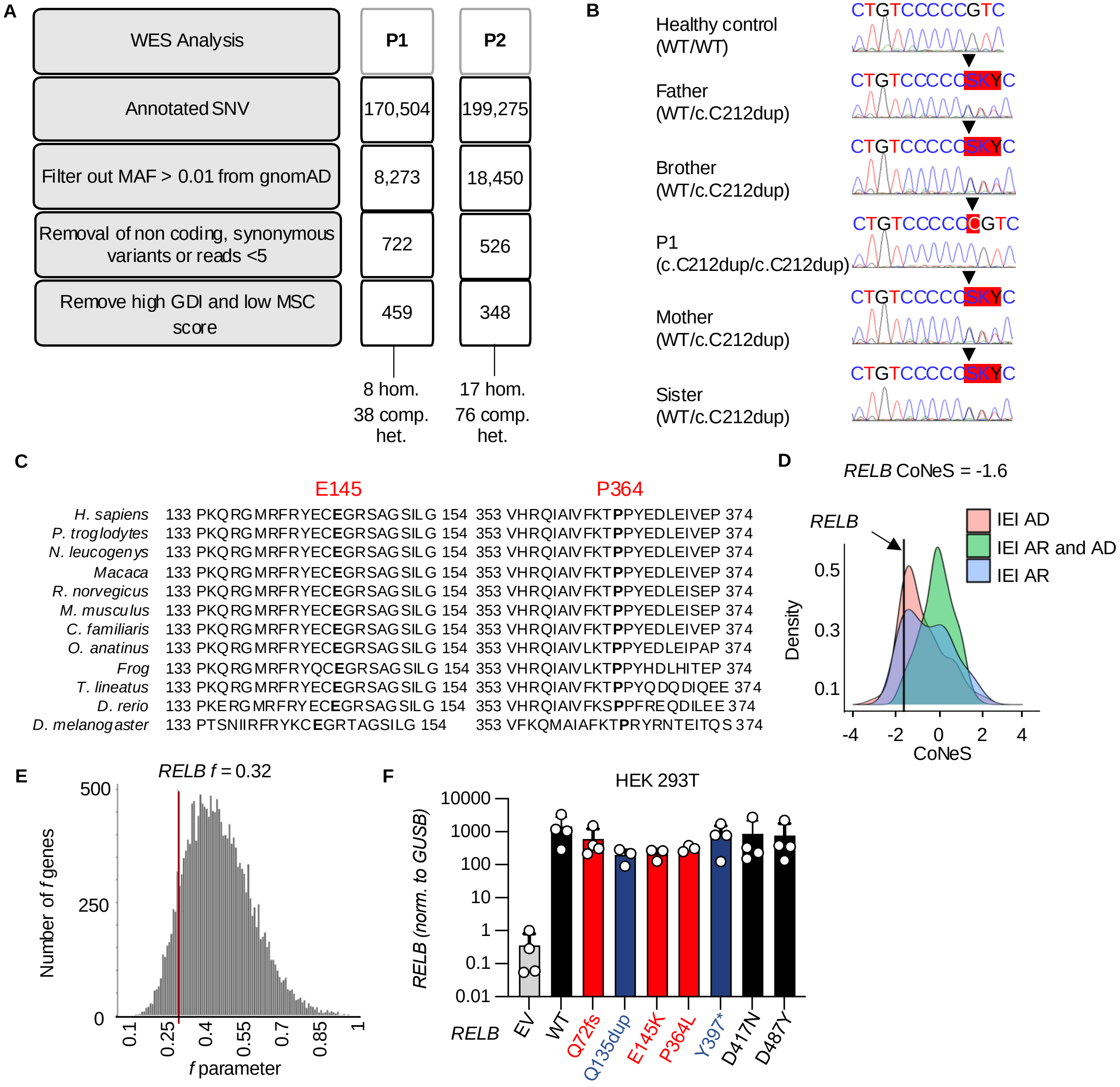

Supplementary Figure 2

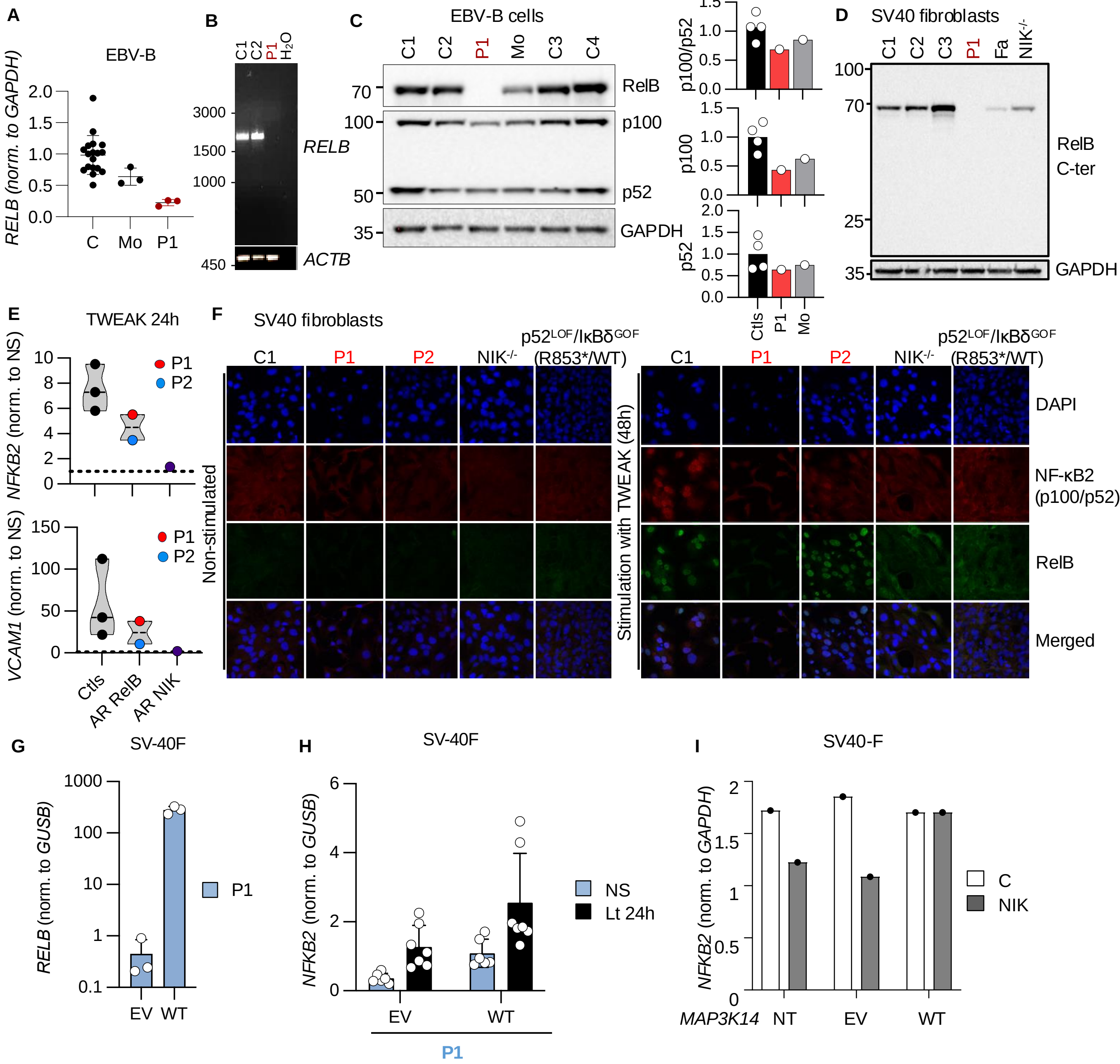

Supplementary Figure 3

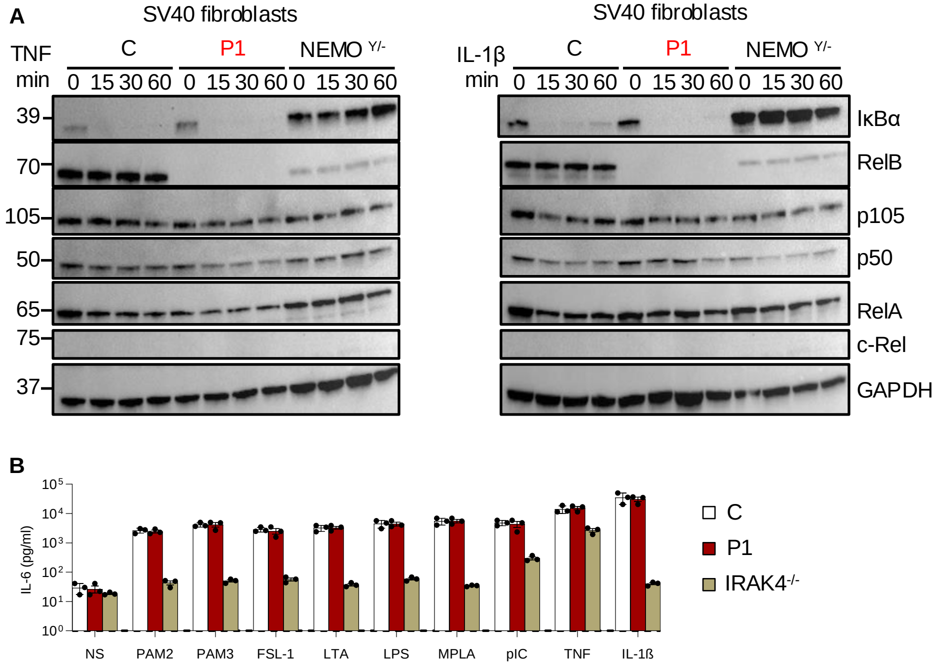

Supplementary Figure 4

Enriched auto-Abs in patients with AR RelB deficiency

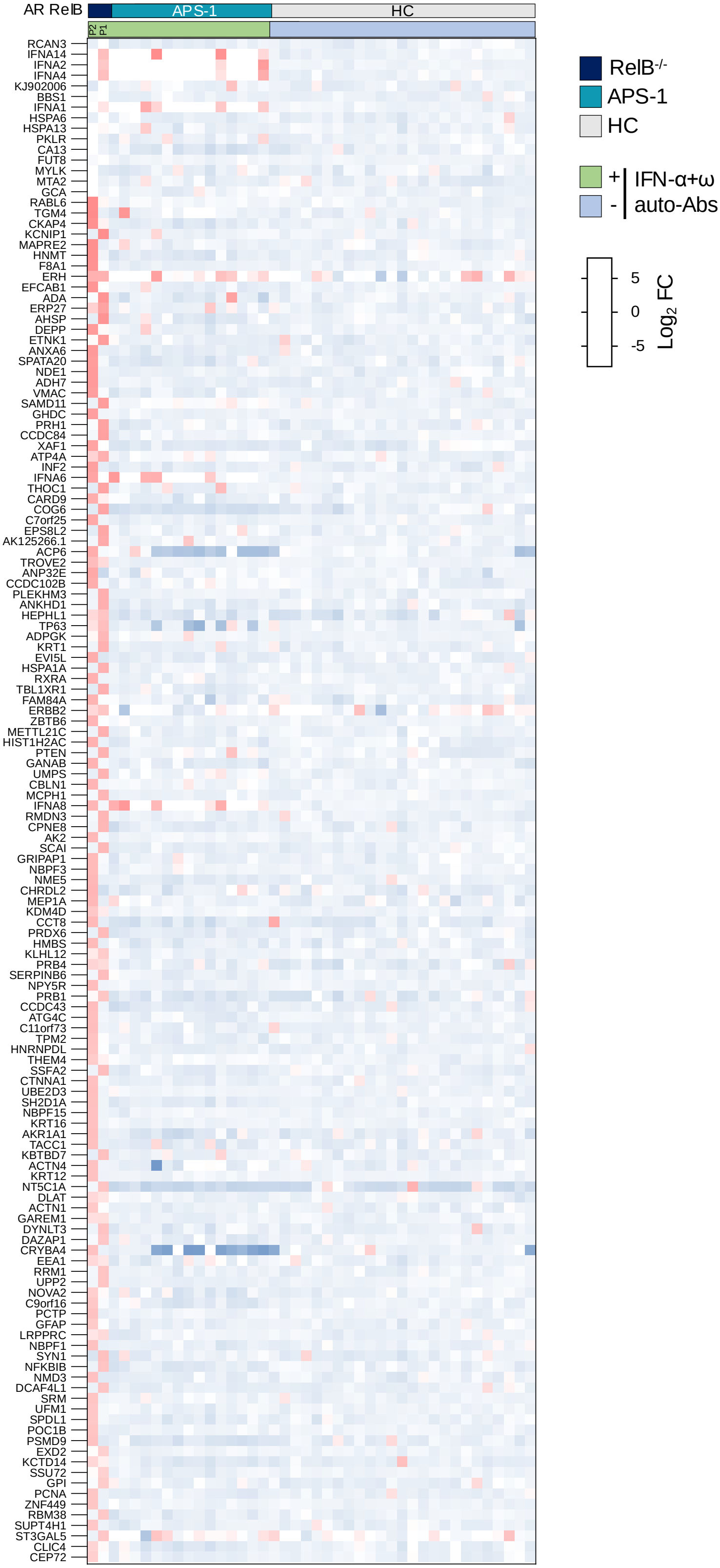

Supplementary Figure 5

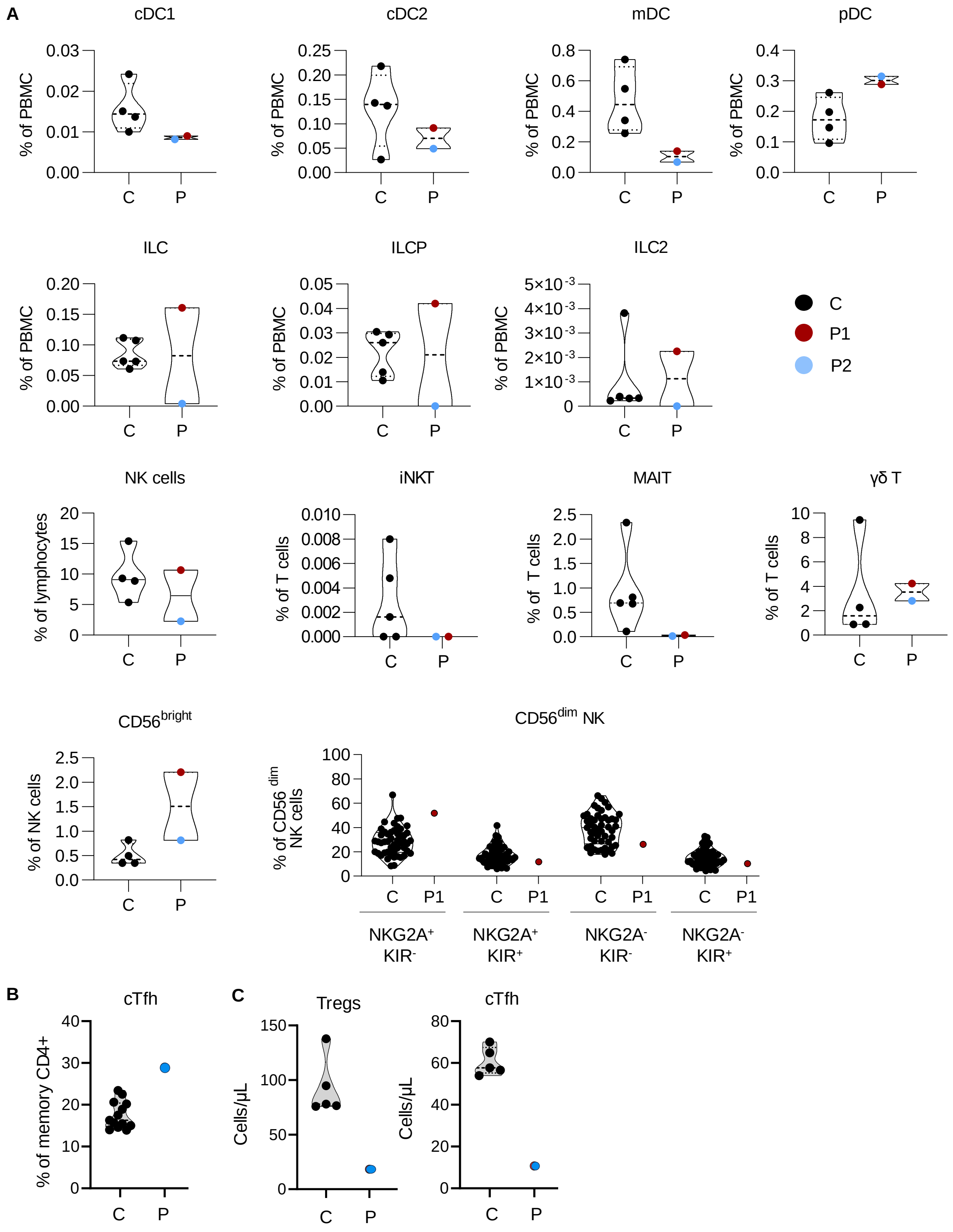

Supplementary Figure 6

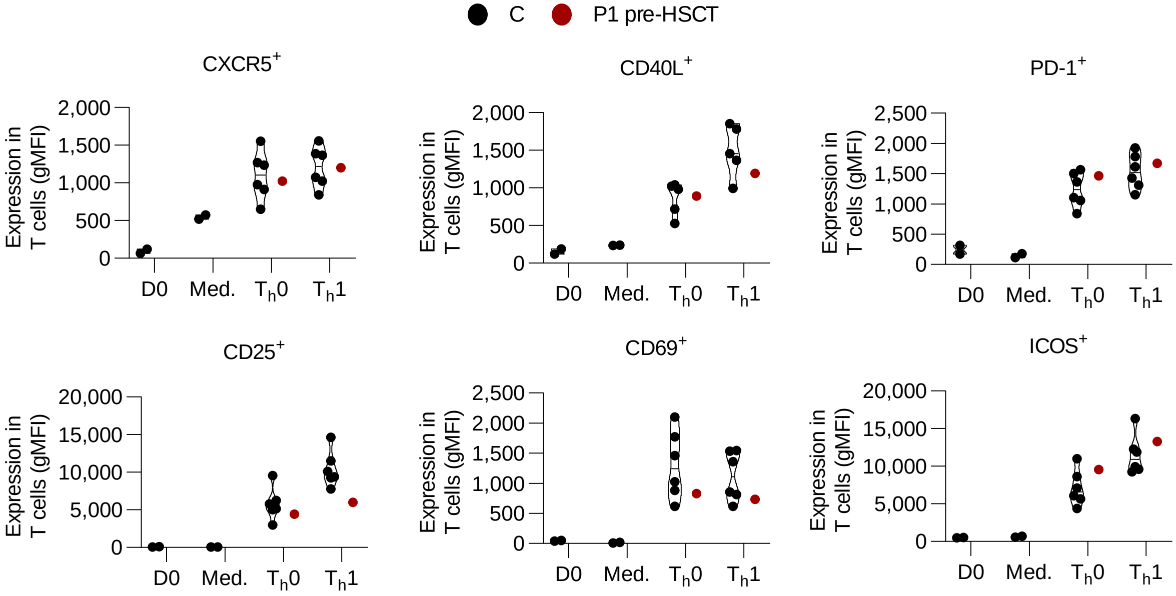

Supplementary Figure 7

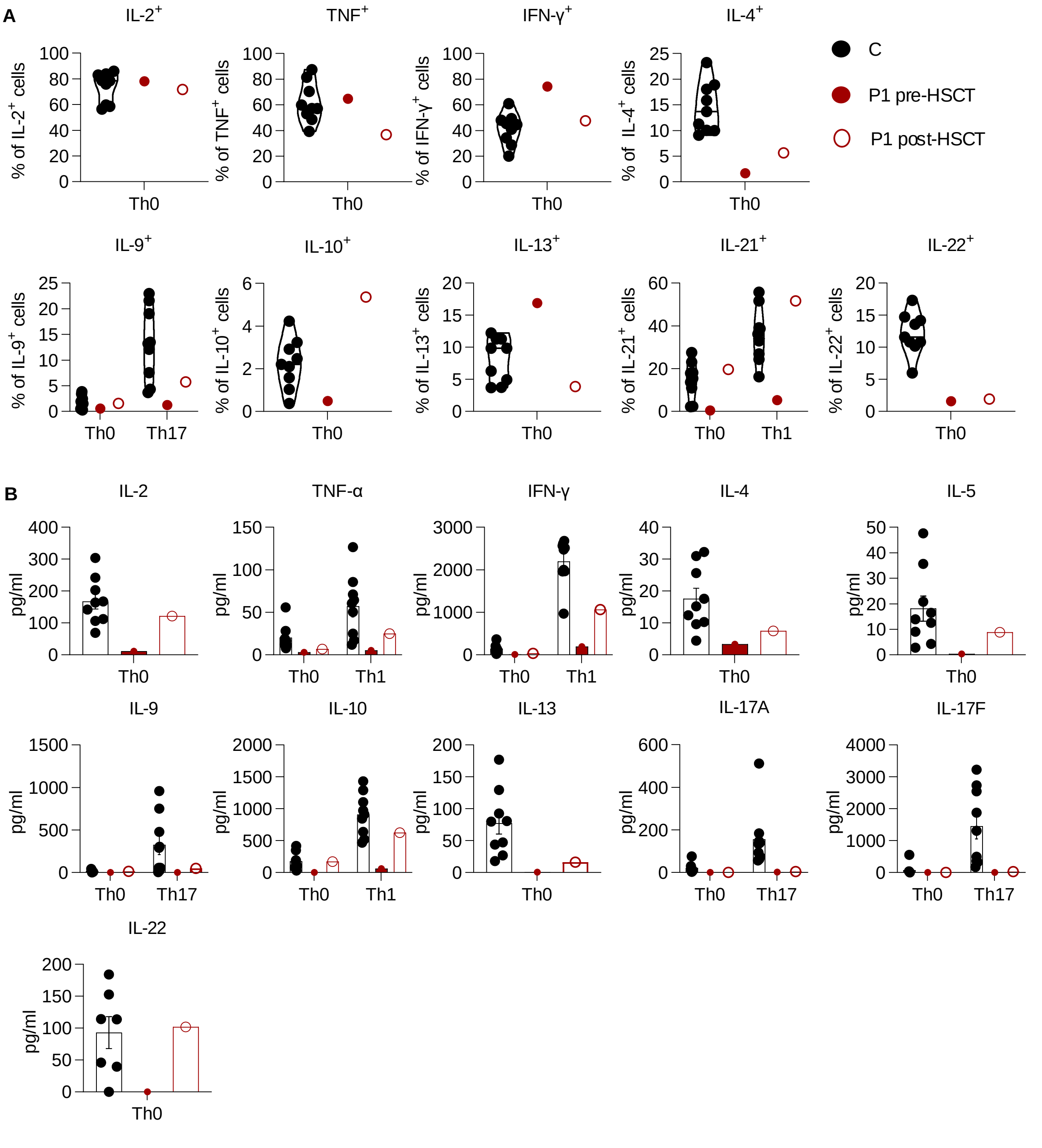

Supplementary Figure 8

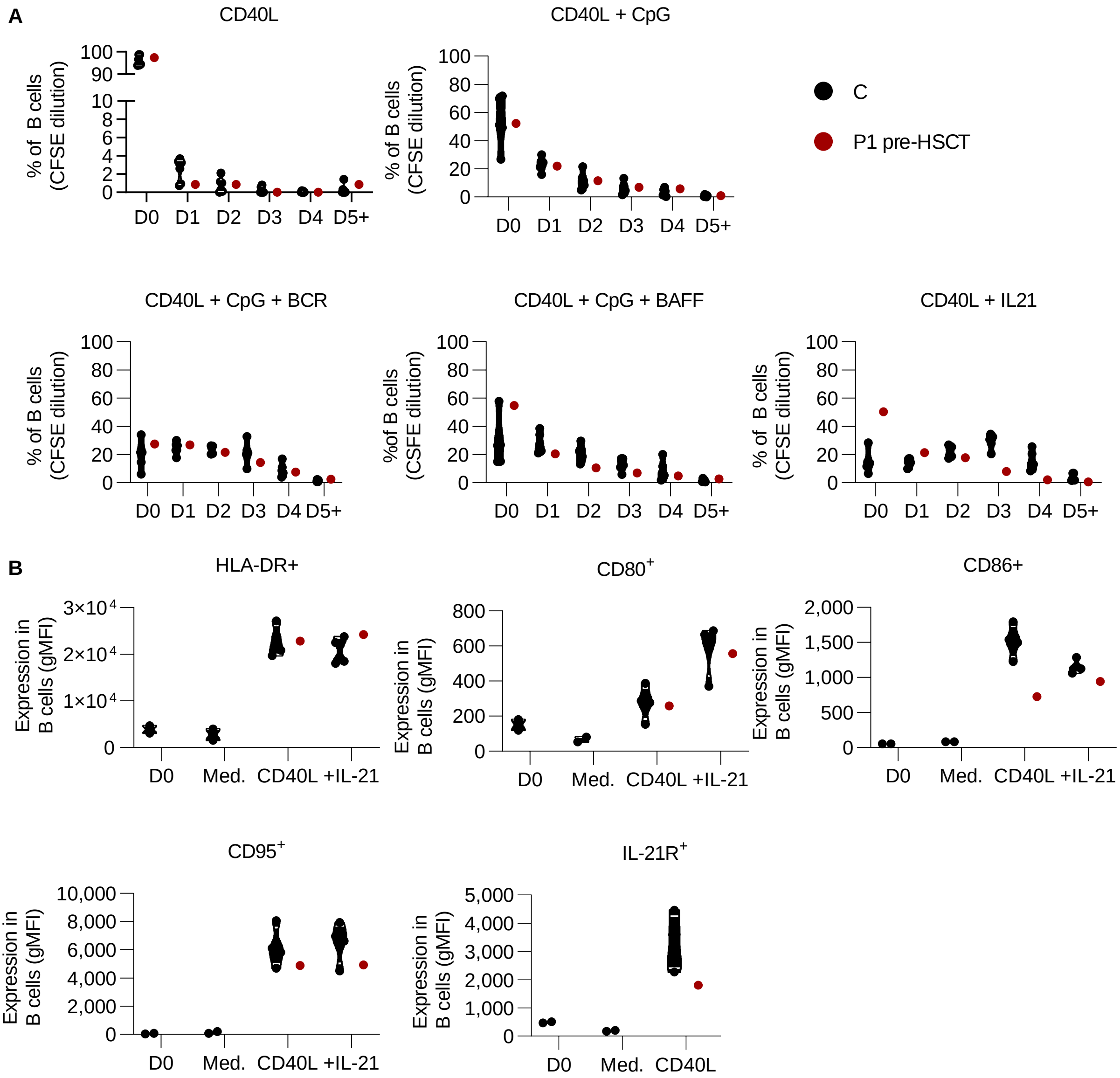

Supplement: Supplementary file 2 — Appendix 02 (PDF) [file pnas.2321794121.sapp2.pdf]
